# Biofilm Producing Rhizobacteria With Multiple Plant Growth-Promoting Traits Promote Growth of Tomato Under Water-Deficit Stress

**DOI:** 10.3389/fmicb.2020.542053

**Published:** 2020-11-26

**Authors:** Md. Manjurul Haque, Md Khaled Mosharaf, Moriom Khatun, Md. Amdadul Haque, Md. Sanaullah Biswas, Md. Shahidul Islam, Md. Mynul Islam, Habibul Bari Shozib, Md. Main Uddin Miah, Abul Hossain Molla, Muhammad Ali Siddiquee

**Affiliations:** ^1^Department of Environmental Science, Faculty of Agriculture, Bangabandhu Sheikh Mujibur Rahman Agricultural University, Gazipur, Bangladesh; ^2^Department of Agro-Processing, Faculty of Agriculture, Bangabandhu Sheikh Mujibur Rahman Agricultural University, Gazipur, Bangladesh; ^3^Department of Horticulture, Faculty of Agriculture, Bangabandhu Sheikh Mujibur Rahman Agricultural University, Gazipur, Bangladesh; ^4^Bangladesh Jute Research Institute, Dhaka, Bangladesh; ^5^Plant Pathology Division, Bangladesh Agricultural Research Institute, Gazipur, Bangladesh; ^6^Grain Quality and Nutrition Division, Bangladesh Rice Research Institute, Gazipur, Bangladesh; ^7^Department of Agroforestry and Environment, Faculty of Agriculture, Bangabandhu Sheikh Mujibur Rahman Agricultural University, Gazipur, Bangladesh

**Keywords:** extracellular polymeric substances, nanocellulose, indole-3-acetic acid, nutrient solubilization, siderophore production, lipid peroxidation, catalase

## Abstract

Plant growth-promoting rhizobacteria (PGPR) not only enhance plant growth but also control phytopathogens and mitigate abiotic stresses, including water-deficit stress. In this study, 21 (26.9%) rhizobacterial strains isolated from drought-prone ecosystems of Bangladesh were able to form air–liquid (AL) biofilms in the glass test tubes containing salt-optimized broth plus glycerol (SOBG) medium. Based on 16S rRNA gene sequencing, *Pseudomonas chlororaphis* (ESR3 and ESR15), *P. azotoformans* ESR4, *P. poae* ESR6, *P. fluorescens* (ESR7 and ESR25), *P. gessardii* ESR9, *P. cedrina* (ESR12, ESR16, and ESR23), *P. veronii* (ESR13 and ESR21), *P. parafulva* ESB18, *Stenotrophomonas maltophilia* ESR20, *Bacillus cereus* (ESD3, ESD21, and ESB22), *B. horikoshii* ESD16, *B. aryabhattai* ESB6, *B. megaterium* ESB9, and *Staphylococcus saprophyticus* ESD8 were identified. Fourier transform infrared spectroscopy studies showed that the biofilm matrices contain proteins, polysaccharides, nucleic acids, and lipids. Congo red binding results indicated that these bacteria produced curli fimbriae and nanocellulose-rich polysaccharides. Expression of nanocellulose was also confirmed by Calcofluor binding assays and scanning electron microscopy. *In vitro* studies revealed that all these rhizobacterial strains expressed multiple plant growth-promoting traits including N_2_ fixation, production of indole-3-acetic acid, solubilization of nutrients (P, K, and Zn), and production of ammonia, siderophores, ACC deaminase, catalases, lipases, cellulases, and proteases. Several bacteria were also tolerant to multifarious stresses such as drought, high temperature, extreme pH, and salinity. Among these rhizobacteria, *P. cedrina* ESR12, *P. chlororaphis* ESR15, and *B. cereus* ESD3 impeded the growth of *Xanthomonas campestris* pv. *campestris* ATCC 33913, while *P. chlororaphis* ESR15 and *B. cereus* ESD21 prevented the progression of *Ralstonia solanacearum* ATCC^®^ 11696^TM^. In a pot experiment, tomato plants inoculated with *P. azotoformans* ESR4, *P. poae* ESR6, *P. gessardii* ESR9, *P. cedrina* ESR12, *P. chlororaphis* ESR15, *S. maltophilia* ESR20, *P. veronii* ESR21, and *B. aryabhattai* ESB6 exhibited an increased plant growth compared to the non-inoculated plants under water deficit-stressed conditions. Accordingly, the bacterial-treated plants showed a higher antioxidant defense system and a fewer tissue damages than non-inoculated plants under water-limiting conditions. Therefore, biofilm-producing PGPR can be utilized as plant growth promoters, suppressors of plant pathogens, and alleviators of water-deficit stress.

## Introduction

Climate change is one of the key global concerns for sustainable agricultural production. According to the Intergovernmental Panel on Climate Change (Intergovernmental Panel on Climate Change [IPCC], 2014), significant areas of fertile agricultural lands will probably be lost or degraded due to adverse effects of climate change (e.g., sea level raising, salinization, heat, and drought) at the end of the 21st century. Another forecasted is that climate change may limit nutrient bioavailability in soils (Karmakar et al., 2016) and a change in the dynamics of pathogen and pest reproduction (Sharma et al., 2017). Furthermore, the climate change may cause the emergence of new pathogens and pests. All these effects will ultimately affect the existing crop production system that extensively relies on chemical fertilizers and pesticides. The problem, however, is that an excessive use of agro-chemicals also causes environmental (in the air, water, and soil) pollution (Jarecki et al., 2008). A climate-friendly agriculture, therefore, depends on how well natural biota are used in the agricultural production systems, particularly in water-limiting conditions (drought).

Bangladesh is one of the most vulnerable countries in the world to climate change, including water-deficit stress. It was reported that in Bangladesh 32.4% of the land during May to October (Kharif season), 27.2% in November to April (Rabi season), and 16.2% between March to May (pre-Kharif season) are under the threat of extreme water shortage, respectively (Alamgir et al., 2019). The scientific community agrees that plant growth and yield is affected by water-deficit stress (Vurukonda et al., 2016; Chandra et al., 2018; Martins et al., 2018; Saikia et al., 2018; Meenakshi et al., 2019). Water-deficit stress aggravates overproduction of reactive oxygen species (ROS) that promote oxidative damage by oxidizing proteins, lipids, nucleic acids, and other cellular macromolecules in plants (Sgherri et al., 2000; Vurukonda et al., 2016). However, plants have an abundant network for ROS detoxification including either enzymatic antioxidants, such as catalases, ascorbate peroxidases, superoxide dismutases, and glutathione reductases or non-enzymatic components through proline, carotenoids, phenolics, and flavonoids (Du et al., 2004; Kaushal and Wani, 2016). In order to combat water-deficit stress, tolerant plant varieties could be used in the future despite the fact that traditional breeding is time consuming. Nevertheless, developing transgenic crops through gene transfer or gene editing using the CRISPR/Cas9 system is associated with ethical and social acceptance issues. In light of these problems, application of beneficial microbes including plant growth-promoting rhizobacteria (PGPR) would be a simple and cheap way for improving plant resiliency against water-deficit stress.

Plant growth-promoting rhizobacteria are root-colonizing beneficial bacteria. They are known to directly enhance plant growth by providing nutrients (e.g., nitrogen, phosphorous, potassium, zinc, and iron) and by producing phytohormones [e.g., indole-3-acetic acid (IAA), cytokinins, gibberellins, and ethylene] or indirectly by synthesizing antibiotics, hydrolytic enzymes (e.g., amylases, cellulases, proteases, lipases, chitinases, pectinases, and dehydrogenases) and volatile compounds (e.g., hydrogen cyanide, ammonia, acetoin, and indole), by inducing systemic resistance, and through biocontrol of phytopathogens (Lugtenberg and Kamilova, 2009; Kumar et al., 2012; Mohite, 2013; Gupta et al., 2017; Backer et al., 2018; Gouda et al., 2018). PGPR are also known for their effectiveness in alleviating water-deficit stress in plants through production of phytohormones and 1-aminocyclopropane-1-carboxylate (ACC) deaminase that reduce ethylene levels in the roots. They also induce systemic tolerance by bacterial compounds, expression of antioxidant enzymes, and production of extracellular polymeric substances (EPS) (Timmusk et al., 2014; Naseem et al., 2018; Niu et al., 2018). To date, numerous PGPR (e.g., *Azospirillum brasilense*, *Bacillus cereus*, *B. subtilis*, *B. amyloliquefaciens*, *B. licheniformis*, *B. thuringiensis*, *Burkholderia* sp., *Citrobacter freundii*, *Paenibacillus polymyxa*, *Proteus penneri*, *Pseudomonas fluorescens*, *P. putida*, *P. aeruginosa*, *Ochrobactrum pseudogrignones*, and *Azotobacter chroococcum*) were identified that promote plant growth under water-deficit stress conditions in greenhouse experiments (Wang et al., 2012; Ngumbi and Kloepper, 2016; Vurukonda et al., 2016; Chandra et al., 2018; Martins et al., 2018; Saikia et al., 2018; Van Oosten et al., 2018; Jochum et al., 2019; Meenakshi et al., 2019). However, screening of bacterial strains for PGPR functions in the laboratory/greenhouse not always results in identifying strains that promote plant growth under field conditions. The failure would be overcome by application of bacterial inoculants in the form of biofilms, thus protecting the inoculants against water-deficit stress (Timmusk et al., 2005; Saleh-Lakha and Glick, 2006; Seneviratne et al., 2010).

Biofilms are surface-associated microbial cells, encased in a self-produced EPS that predominantly contain proteins, polysaccharide, extracellular DNA, and lipids (Flemming and Wingender, 2010). The literature contains several examples of biofilm PGPR that are much more effective under field conditions than any planktonic PGPR (Seneviratne et al., 2010; Pandin et al., 2017; Backer et al., 2018). Zhang et al. (2015) reported that nitrogenase activity, IAA production, phosphate solubilization, siderophore production, and ammonia production are incredibly higher in biofilm PGPR than the planktonic PGPR. Other advantages of biofilm PGPR are their higher resistance to antibiotics and adverse environmental stresses (e.g., high temperature, extreme pH, salinity, and drought), leading to an improved chance of survival in a competitive soil environment (Mah et al., 2003). Biofilm PGPR also produce remarkably higher amounts of antimicrobial compounds than the planktonic PGPR, leading to suppression of phytopathogens (Pandin et al., 2017). However, quite a few biofilm PGPR (e.g., *Rhizobium leguminosarum*, *Agrobacterium* sp., *A. vinelandii*, *Enterobacter cloacae*, *Xanthomonas* sp., *Pseudomonas* sp., *P. polymyxa*, *Bradyrhizobium* sp., *Bacillus subtilis*, and *B. drentensis*) were identified till date (Bais et al., 2004; Timmusk et al., 2005; Ude et al., 2006; Mahmood et al., 2016; Gupta et al., 2017). Thus, biofilm-producing bacteria could be advantageous over others to thrive in a new water-stress environment for delivering beneficial effects to plants. However, biofilm-producing bacteria associated with crop species which are naturally adapted to water stress, such as tomato, have not been explored so far.

Tomato (*Solanum lycopersicum* L.) is one of the most popular vegetables cultivated worldwide. It is rich in micronutrients, antioxidants, phenolics, flavonoids, vitamins, and essential trace elements. Currently, water stress limits the productivity of tomato worldwide, including Bangladesh (Habiba and Shaw, 2013). This study reports the results of a series of experiments we carried out to screen and identify biofilm-producing bacteria from the tomato rhizosphere grown in water stress-prone areas (Rajshahi, Dinajpur, and Bogura districts) of Bangladesh. We also characterized the matrix components, i.e., EPS, generated by different bacterial biofilms by means of Fourier transform infrared spectroscopy, scanning electron microscopy, and different binding assays. The expressions of multiple PGP- and biocontrol-related traits in these bacteria were examined *in vitro*. Moreover, selected biofilm-producing rhizobacterial strains were evaluated *in vivo* for their tomato growth promotion under water-deficit stress conditions. This study contributes toward an understanding about how biofilm-producing rhizobacteria may help to promote plant growth, suppress phytopathogens, and reduce water-deficit stress.

## Materials and Methods

### Collection of Rhizospheric Soil Samples and Isolation of Bacteria

Tomato plants (*Solanum lycopersicum* L.) were uprooted on soil during the flowering stage collected from three drought-prone areas of Bangladesh, including Rajshahi, Dinajpur, and Bogura. Geographical positions (GPS) of the sampling areas are shown in Table 1. Each sample (excluding the aboveground parts) was transferred into a sterile polythene bag and then transported to the laboratory. During transportation, the cold chain was maintained by keeping the samples in an icebox. The collected samples were stored at 4°C before the isolation of bacteria. In order to isolate rhizobacteria, firstly non-rhizosphere soil was discarded by hand shaking. Then, the sample (roots together with soil) was homogenized using a sterile mortar pestle in sterile distilled water, and a serial dilution was performed. After the serial dilution, 50 μL of each sample was spread on yeast extract peptone [YEP (1% of peptone, 0.5% of yeast extract, pH 6.8)] agar (1.5%) plates and incubated at 28°C in a stationary condition. After a 36-h incubation, morphologically distinct (e.g., in size, shape, and color) colonies were transferred to fresh YEP agar plates using sterile toothpicks. In order to prepare pure cultures, the repeated-streaking method was followed.

**TABLE 1:** Identification of biofilm-producing rhizobacteria isolated from tomato rhizosphere grown in water stress-prone ecosystems of Bangladesh.

Strains	Geographical position	Length (bp)	Maximum score	Identity (%)	Identified as	Accession number	Gene bank accession
Rajshahi							
ESR3	24.03814 N; 90.39731 E	1405	2575	99.72	*Pseudomonas chlororaphis*	AJ550465.1	MN180835
ESR4	24.42656 N; 88.42353 E	1403	2591	100	*P. azotoformans*	MK883104.1	MN173418
ESR6	24.42941 N; 88.40231 E	1383	2359	97.47	*P. poae*	MK883118.1	MN173419
ESR7	24.43090 N; 88.40452 E	1412	2580	99.65	*P. fluorescens*	KT767924.1	MN173420
ESR9	24.44068 N; 88.40942 E	1414	2584	99.65	*P. gessardii*	MG972901.1	MN173421
ESR12	24.44067 N; 88.40943 E	1414	2603	99.93	*P. cedrina*	KT767922.1	MN173422
ESR13	24.44115 N; 88.41311 E	1408	2584	99.79	*P. veronii*	MH665745.1	MN180836
ESR15	24.44416 N; 88.41007 E	1415	2603	99.93	*P. chlororaphis*	AJ550465.1	MN173423
ESR16	24.03814 N; 90.39732 E	1402	2590	100	*P. cedrina*	KT767922.1	MN173424
ESR20	24.42657 N; 88.42353 E	1424	2603	99.72	*Stenotrophomonas maltophilia*	KM893074.1	MN173425
ESR21	24.43091 N; 88.40943 E	1416	2608	99.93	*P. veronii*	MH665745.1	MN173426
ESR23	24.44068 N; 88.40944 E	1407	2593	99.93	*P. cedrina*	KT767660.1	MN173427
ESR25	24.44419 N; 88.41009 E	1405	2588	99.93	*P. fluorescens*	KY670742.1	MN180837
Dinajpur							
ESD3	25.48203 N; 88.86574 E	1429	2582	99.30	*Bacillus cereus*	JQ799048.1	MN173428
ESD8	25.48393 N; 88.86528 E	1429	2639	100	*Staphylococcus saprophyticus*	MK841545.1	MN173429
ESD16	25.48452 N; 88.86705 E	1408	2374	97.09	*B. horikoshii*	KJ534599.1	MN173430
ESD21	25.48635 N; 88.86585 E	1422	2569	99.30	*B. cereus*	JQ799048.1	MN173431
Bogura							
ESB6	24.5136 N; 89.2218 E	1427	2636	100	*Bacillus aryabhattai*	MN004848.1	MN173432
ESB9	24.5130 N; 89.2242 E	1431	2438	99.93	*B. megaterium*	KC692173.1	MN173433
ESB18	24.5131 N; 89.2229 E	705	1242	98.44	*P. parafulva*	MT089718.1	MT448933
ESB22	24.5128 N; 89.2241 E	1429	2639	100	*B. cereus*	MG937670.1	MN173434

### Selection of Biofilm-Producing Rhizobacteria

To screen biofilm-producing rhizobacteria, each strain was initially inoculated in YEP broth and incubated at 28°C under agitating conditions (160 rpm) for 6 h [until the optical density (OD_660_) reached 0.6 to 0.8]. Then, 1 mL culture of each strain was collected and centrifuged at 12000 rpm for 10 min. The supernatant was carefully discarded. The pellet was resuspended in sterile distilled water and then diluted (ca. 10^5^ colony-forming units (CFU) mL^–1^)]. Afterward, 50-μL cultures were inoculated into glass test tubes (Pyrex, flat bottom, Glassco, United Kingdom) containing 5 mL salt-optimized broth plus glycerol (SOBG) medium (per liter: 20 g of tryptone, 5 g of yeast extract, 0.5 g of NaCl, 2.4 g of MgSO_4_⋅7H_2_O, 0.186 g of KCl, and 50 mL of 40% glycerol, pH 7.0) and incubated at 28°C under static conditions. After 72 h of incubation, air–liquid (AL) and/or solid–air–liquid (SAL) biofilm-producing rhizobacteria were selected as described in Haque et al. (2012, 2015), and photographs were taken. The biomass of biofilms and the enumeration of bacterial cells coupled with biofilm matrices were determined as described in Mosharaf et al. (2018).

### 16S rRNA Gene Sequencing

The protocol described by Sambrook et al. (1989) was used for the collection of bacterial DNA. The 16S rRNA gene primers of 27F (5′-AGAGTTTGATCMTGGCTCAG-3′) and 1492R (5′-GGTTACCTTGTTACGACTT-3′) were used to amplify the 16S rRNA gene by polymeric chain reaction (PCR). To amplify the gene by PCR, the following conditions were set: initial DNA denaturation for 5 min at 94°C, 35 cycles of denaturation for 30 s at 94°C, annealing for 45 s at 57°C, elongation for 1.5 min at 72°C, and a final extension for 10 min at 72°C. The QIAquick^®^ Gel Extraction Kit (Qiagen, GmbH, Germany) was used to purify the PCR products and then sequenced by using 3500 Genetic Analyzer (Applied Biosystems). Using the BLASTN (Basic Local Alignment Search for Nucleotide) program, we compared the gene sequences of different bacterial strains against the sequences of bacteria available in NCBI (National Center for Biotechnology Information) data banks^1^. We deposited the obtained 16S rRNA gene sequences in the GenBank nucleotide databases under accession numbers from MN173418 to MN173434, MN180835 to MN180837, and MT448933^2^.

### Identification of the Matrix Components of the Biofilms

Fourier transform infrared (FTIR) spectroscopy, scanning electron microscopy (SEM), and Congo red and Calcofluor binding assays were used to identify the components of the biofilm matrices, i.e., EPS. For the FTIR analysis, the pellets were prepared as described in Mosharaf et al. (2018). Using the triglycine sulfate (TGS) detector, 450 to 4000 cm^–1^ was scanned (16 scans at 4 cm^–1^ resolution and at 0.2 cm sec^–1^ scanning speed). The IR spectra of the biofilm matrices were acquired using the Perkin Elmer FTIR (Spectrum-2) instrument operated by CPU32M software. Perkin Elmer’s proprietary software (Version 10.05.03) was used to analyze the baseline subtracted biofilm spectra. For SEM, 72-hour-old biofilms were carefully collected and then oven dried at 40°C for 48 h. Each dried sample was coated with carbon using a vacuum sputter-coater to improve the conductivity. A scanning electron microscope (SEM, JEOL JSM-6490LA, Japan) operated at 5.0 KV was used to image the samples. To detect curli fimbriae and nanocellulose, Congo red and Calcofluor binding assays were performed as described in Haque et al. (2009, 2017).

### Assessment of the *in vitro* Plant Growth Promotion Activities

#### Indole-3-Acetic Acid Production

Production of indole-3-acetic acid (IAA) was detected as described by Gordon and Weber (1951) with a few modifications. In brief, one single colony of each rhizobacterium was inoculated in 5 mL Luria-Bertani (LB) broth (1% of tryptone, 0.5% of yeast extract, 0.5% of NaCl, pH 7.0) supplemented with 0.2% of L-tryptophan (Bio Basic Inc., Canada) and incubated at 28°C under shaking conditions (160 rpm). After a 48-hour incubation, 1 mL culture was collected and centrifuged at 14000 rpm for 10 min. Then, 500 μL supernatant was transferred to a sterile glass test tube and mixed with 1 mL Salkowski reagent (98 mL 35% perchloric acid and 2 mL 0.5 M FeCl_3_). The test tubes were incubated at room temperature for 45 min in the dark. Development of pink color indicated positive for auxin production. Among the auxins, IAA (μg mL^–1^) production was quantified spectrophotometrically at OD_5__3__0_ nm and compared to a standard curve prepared from commercial IAA (Duchefa Biochemie, Netherlands) with a concentration range of 0 to 1000 μg mL^–1^.

Colorimetric IAA results were also verified and quantified using high-performance liquid chromatography (HPLC). In brief, all the rhizobacterial strains were grown as described in the colorimetric determination section. The cell-free extract was prepared by centrifugation followed by filtration through Einmalfilter (CHROMAFIL^®^ Xtra PTFE-45/25, 0.45 μM, Macherey-Nagel, GmbH and Co. KG, Germany). The cell-free extract was acidified (2.5 to 3) using 1 N HCl and extracted with ethyl acetate (1:1 v/v), then the organic phase was collected. A rotary evaporator was used to evaporate the ethyl acetate fraction. The crude extract was dissolved in methanol (HPLC grade, Sigma-Aldrich, St. Louis, MO, United States) and kept at 4°C for further use. In order to detect IAA specifically, Shimadzu Prominence HPLC (Japan) using a C18 analytical column (4.6 mm × 250 mm, 5 μm) was used. The column temperature was maintained at 25°C, and methanol and 1% acetic acid (50:50 v/v) were used as the mobile phase at a flow rate of 1 mL min^–1^ with an injection volume of 20 μL (Myo et al., 2019). Detection was monitored at 254 and 280 nm, and data were evaluated using Lab Solutions software by comparing with the elution profiles of standard IAA (6.25, 12.5, 25, 50, and 100 μg mL^–1^) injected separately.

#### Ability of Nitrogen Fixation

Nitrogen fixation assays were performed according to Ker (2011) with minor modifications. In brief, initially, each rhizobacterium strain was grown in YEP broth under shaking conditions (120 rpm) at 28°C for 16 h, then diluted to 10^7^ CFU mL^–1^. Two microliters (2 μL) of diluted culture of each rhizobacterium strain was spotted (4 spots plate^–1^) onto N-free solid LG agar (per liter: 10 g sucrose, 0.5 g K_2_HPO_4_, 0.2 g MgSO_4_⋅7H_2_O, 0.2 g NaCl, 0.001 g MnSO_4_⋅H_2_O, 0.001 g FeSO_4_, 0.001 g Na_2_MoO_4_⋅2H_2_O, 5 g CaCO_3_, and 15 g agar) plates and incubated at 28°C under static conditions for 10 days. For control, non-inoculated N-free solid LG agar plates were incubated at the same condition. Development of colonies indicated a positive nitrogen fixation.

The dinitrogenase reductase gene, *nifH*, the most widely used biomarker for the study of nitrogen-fixing bacteria, was analyzed by PCR. In this study, the primer pair of Ueda19F-GCIWTYTAYG GIAARGGIGG and Ueda19R-AAICCRCCRCAIACIACRTC was used to amplify a 389-bp *nifH* fragment (Ueda et al., 1995), and that of KAD3F-ATHGT IGGITGYGAYC CIAARGCIGA and DVVR-ATIGCRAAICCI CCRCAIACIACRTC was used to amplify a 310-bp *nifH* fragment (Ando et al., 2005). The reaction conditions with 100 ng of template DNA were as follows: one cycle at 95°C (5 min); 30 cycles at 95°C (30 s), 51°C for Ueda19F and Ueda19R or 56°C for KAD3F and DVVR (30 s) and 72°C (1 min); plus one cycle at 72°C (7 min). For negative control, the water was used instead of DNA.

#### Phosphate Solubilization

Qualitative phosphate (P) solubilization ability was assessed as described in Nautiyal (1999). In brief, 2 μL of overnight-grown culture (ca. 10^7^ CFU mL^–1^) was speckled (4 spots plate^–1^) onto National Botanical Research Institute’s phosphate (NBRIP) agar [per liter: 10 g glucose, 5 g MgCl_2_⋅6H_2_O, 0.25 g MgSO_4_⋅7H_2_O, 0.2 g KCl, 0.1 g (NH_4_)_2_SO_4_, and 15 g agar] plates containing 0.5% Ca_3_(PO_4_)_2_ or 0.8% rock phosphate. For control, each bacterial strain was dotted onto Ca_3_(PO_4_)_2_/rock phosphate-free NBRIP agar plates. The inoculated plates were incubated at 28°C under static conditions for 96 h. The appearance of a clearing zone around the colonies indicated a positive phosphate solubilization. The following equation was used to calculate the phosphate solubilization index (PSI):

$$PSI=\frac{Diameterofbacterialcolony+Diameterofhalozone}{Diameterofbacterialcolony}$$

#### Potassium Solubilization

To screen for K-solubilizing rhizobacteria, 2 μL culture (ca. 10^7^ CFU mL^–1^) of each bacterial strain was spotted (4 spots plate^–1^) onto Aleksandrov agar medium [per liter: 5.0 g glucose, 0.5 g MgSO_4_⋅7 H_2_O, 0.005 g ferric chloride, 0.1 g calcium carbonate, 2 g calcium phosphate, 2 g potassium aluminum silicate (as a source of insoluble inorganic potassium), and 15 g agar]. For control, each bacterial strain was marked onto potassium aluminum silicate-deficient Aleksandrov agar plates. Inoculated plates were incubated at 28°C for 7 days. The appearance of a clearing zone around the colonies indicated a positive potassium solubilization.

#### Zinc Solubilization

Zinc (Zn) solubilization assays were done as described in Saravanan et al. (2007) with a few modifications. In brief, 2 μL (ca. 10^7^ CFU mL^–1^) culture of each rhizobacterium strain was spotted (4 spots plate^–1^) onto the basal agar medium (per liter: 10 g glucose, 1 g (NH_4_)_2_SO_4_, 0.2 g KCl, 0.1 g K_2_HPO_4_, MgSO_4_⋅7H_2_O, and 15 g agar, pH 7.0) containing 0.2% insoluble Zn from three sources, such as ZnO, ZnCO_3_, and Zn_3_(PO_4_)_2_ (Wako Pure Chemical Industries Ltd., Japan). For control, the bacterial strain was spotted onto the Zn-free agar plates. The inoculated plates were kept at 28°C under static conditions. A clearing zone around the colonies indicated positive results. The Zn solubilization index (ZnSI) was also calculated after 7 days using the same equation as for PSI. 

#### Production of Siderophores

Overlay chrome azurol S (O-CAS) medium was used to detect siderophores (Shin et al., 2001). Initially, each bacterial strain was grown in YEP broth under shaking conditions at 28°C until OD_660_ reached 0.6–0.8. Then, 2 μL (ca. 10^7^ CFU mL^–1^) diluted culture of each rhizobacterium strain was spotted (1 spot plate^–1^) onto the center of the LB agar plates and incubated at 28°C for 20 h. Later, 10 mL O-CAS broth was applied over those LB agar plates and incubated at 28°C under static conditions. Development of colors (e.g., purple, orange, or yellow) indicated positive results. After a 10-hour incubation, photographs were taken.

#### Production of Volatile Compounds

##### Acetoin

Qualitative acetoin production was examined as described in Dye (1968). In brief, each bacterial culture (16-h old) was inoculated in 5 mL yeast extract salt broth (per liter: 0.5 g of NH_4_H_2_PO_4_, 0.2 g of MgSO_4_⋅7H_2_O, 5.0 g of NaCl, and 5.0 g of glucose) and incubated at 28°C. After a 96-h incubation, 1 mL culture of each bacterium strain was transferred to a sterile glass test tube and 600 μL 5% (w/v) alpha-napthol in absolute alcohol was added and shaken gently. Occurrence of a crimson to ruby color at the top or throughout the mixtures within 4 h indicated a positive acetoin production.

##### Indole

Qualitative indole production was assessed as described in Lelliott and Dickey (1984). Initially, the broth (per liter: 10 g of tryptone, 1 g of L-tryptophan, and an adequate amount of distilled water) was prepared and autoclaved. Then, 50 μL culture (16-hour old) was inoculated in glass test tubes containing 5 mL broth and incubated at 28°C under shaking conditions (160 rpm). After a 96-hour incubation, 500 μL Kovac’s reagent (HiMedia, India) was added to the culture and shaken gently. The development of a dark-red color on the surface of the medium indicated a positive indole production.

##### Ammonia

Qualitative ammonia production was examined by the method described in Dinesh et al. (2018) with a few modifications. In brief, each bacterial strain was grown in YEP broth under shaking conditions for 16 h at 28°C. Then, 50 μL (10^8^ CFU mL^–1^) culture of each bacterium strain was inoculated in glass test tubes containing 5 mL peptone water (Sigma-Aldrich, St. Louis, MO, United States) and incubated at 28°C. After a 72-h incubation, 1 mL Nessler’s reagent (Sigma-Aldrich, St. Louis, MO, United States) was added. The development of yellow to brown color indicated a positive ammonia production.

##### Hydrogen Cyanide (HCN)

Each bacterial colony was streaked on LB agar plates containing 0.45% glycine. Two sterilized filter papers were soaked in alkaline picrate solution (0.25% picric acid in 1.25% sodium carbonate) (Lorck, 1948) and then placed on the lids of petri plates. The plates were sealed with parafilm and incubated at 28°C under static conditions for 24 h. Positive HCN production resulted in a color development on the filter papers from yellow to a reddish-brown.

#### Production of Hydrolytic Enzymes

Qualitative ACC deaminase activity was analyzed as described in Dworkin and Foster (1958) and in Penrose and Glick (2003). In brief, each bacterial strain was grown in LB broth until OD_660_ reached 0.6 to 0.8 and then diluted (ca. 10^7^ CFU mL^–1^). Afterward, 100 μL suspension of each bacterium was spread on minimal DF (Dworkin and Foster) salt agar plates [per liter: 4.0 g KH_2_PO_4_, 6.0 g Na_2_HPO_4_, 0.2 g MgSO_4_⋅7H_2_O, 2.0 g glucose, 2.0 g gluconic acid, 2.0 g citric acid, 1 mg FeSO_4_⋅7H_2_O, 10 mg H_3_BO_3_, 11.19 mg MnSO_4_⋅H_2_O, 124.6 mg ZnSO_4_⋅7H_2_O, 78.22 mg CuSO_4_⋅5H_2_O, 10 mg MoO_3_, 3 mM ACC (nitrogen source), and 1.8% bacto agar]. As a negative control, 100 μL suspension was spread onto ACC-deficient minimal DF salt agar plates. The plates were incubated at 28°C for 4 days. Colonies formed on the plates were considered as ACC-deaminase producers. Qualitative catalase, oxidase, gelatinase, and arginine dihydrolase production was determined as described in Hayward (1992); Shekhawat et al. (1992), Schaad (1988), and Thornley (1960), respectively. Lipase, cellulase, and protease assays were performed as described in Dinesh et al. (2018).

#### Abiotic-Stress Tolerance

The maximum drought stress was reported to be achieved by adding 25% polyethylene glycol (PEG) 6000 into the broth (Vardharajula et al., 2011). Thus, for drought-tolerance stress, all the bacterial strains were grown in LB broth containing 25% PEG 6000 at 28°C under shaking conditions. After a 24-h incubation, the optical density was measured by a spectrophotometer. A bacterial OD_660_ ≥ 0.1 was considered as drought tolerance. Biofilm-producing rhizobacterial strains were also tested for their ability to grow on LB agar plates at different temperatures (37, 42, and 50°C), varying pHs (pH 4.0, 7.0, 8.0, 9.0, and 10.0), and different salinities (5, 10, and 20% NaCl). For control, uninoculated plates/test tubes were incubated. Formation of bacterial colonies on the agar plates under the tested conditions after 72 h was recognized as positive for the respective test.

#### *In vitro* Antagonistic Activities Against Pathogenic Bacteria

All these rhizobacteria strains were tested for their antagonistic activities against *Xanthomonas campestris* pv. *campestris* ATCC 33913 (causal agent of bacterial leaf spot in tomato), *Ralstonia solanacearum* ATCC^®^ 11696^TM^ (causal agent of wilt in tomato), and *Pectobacterium carotovorum* subsp. *carotovorum* PCC8 (causal agent of soft rot in tomato, accession number KX098362) as described in Furuya et al. (1997). In brief, a single colony of each rhizobacterium was spotted onto an LB agar plate and incubated at 28°C under static conditions for 36 h. Then, a sheet of sterilized filter paper was soaked in chloroform and then placed in each lid of the Petri dish and incubated at room temperature for 2 h to kill the rhizobacterium. Then, 5 mL melted water agar (1.5% at 50°C) containing a suspension of each plant pathogenic bacterium [ca. 10^8^ CFU mL^–1^] was poured onto the plates and incubated at 28°C for 48 h. For control, only 5 mL melted water agar (1.5% at 50°C) was poured. The formation of an inhibition zone around the growing region of rhizobacterium was considered positive for the biocontrol agent.

#### Pot Experiment

##### Raising of seedling and characterization of pot soil

Tomato seeds (variety: BARI Tomato 2) were collected from the Horticulture Research Centre (HRC) of Bangladesh Agricultural Research Institute (BARI), Gazipur, Bangladesh. Seeds were surface-sterilized using 5% NaOCl for 2 min then washed 5 times with sterile distilled water. The disinfected seeds were sown in a seedbed (plastic tray containing sterilized soil). The soil was autoclaved twice (121°C, 15 PSI, 1, 24 h pause between cycles) as described by Mahmoudi et al. (2019). For pot experiments, sandy loam soil was collected and properly mixed with decomposed cow dung (3:1). The soil containing cow dung hereafter referred to as soil was then air-dried and sieved (2 mm size sieve). Each pot (height × diameter = 23 cm × 80 cm) was filled with 8 kg (air-dried sieved) soil. The soil pH, organic matter, total N, total P, and exchangeable K were determined as the protocols described in McLean (1982), Nelson and Sommers (1982), Bremner and Mulvaney (1982), Sommers and Nelson (1972), and Barker and Surh (1982), respectively. The pH, organic matter content (%), total N (%), total P (mg kg^–1^), exchangeable K (mg kg^–1^), and bacterial population (CFU g^–1^ soil) (Khan et al., 2017) were found to be 7.27, 2.03, 0.0869, 194.2, 109.4, and 1.6 × 10^3^ (2.63 × 10^7^ without air-drying of the soil–cow dung mixture, hereafter referred to as wet conditions), respectively. In cow dung (air-dried), organic matter content (%), total N (%), total P (mg kg^–1^), and total K (mg kg^–1^) were detected to be 16.65, 1.22, 2100, and 5000, respectively. Total P and total K content in cow dung were determined by the acid digestion method (Jones and Case, 1990; Watson and Isaac, 1990).

##### Experimental design and treatments

The pot experiment was laid out in a complete randomized design (CRD) having nine treatments with four replications. The treatments were T_1_ (applied only a standard dose of N:P:K but no bacteria were inoculated, herein termed as non-inoculated control), T_2_ (*Pseudomonas azotoformans* ESR4), T_3_ (*P. poae* ESR6), T_4_ (*P. gessardii* ESR9), T_5_ (*P. cedrina* ESR12), T_6_ (*P. chlororaphis* ESR15), T_7_ (*Stenotrophomonas maltophilia* ESR20), T_8_ (*P. veronii* ESR21), and T_9_ (*Bacillus aryabhattai* ESB6). Information regarding these bacterial strains on promotion of plant growth and alleviation water-deficit stress was not available in the literature. Therefore, these bacterial strains were selected for this study. The standard dose of N:P:K (as a source of urea, triple super phosphate, and murate of potash, respectively) for the high-yield goal of tomato were calculated using the model of Bangladesh Agricultural Research Council (Fertilizer Recommendation Guide, 2012).

##### Root bacterization, seedling transplantation, and imposing water-deficit stress

For root bacterization, selected rhizobacterial strains were grown in 100 mL YEP broth at 28°C in agitated conditions (150 rpm) until OD_660_ reached 0.6 to 0.8. Then, 1 mL culture of each bacterial strain was harvested and centrifuged at 12000 rpm for 10 min. The supernatant was discarded. The pellets were resuspended in phosphate buffer (pH 7.0) and diluted to ca. 10^8^ CFU mL^–1^. Healthy-looking, uniform-sized seedlings (20-day-old) were carefully uprooted. Afterward, the roots of the seedlings were immersed in bacterial suspension treatment wise (i.e., T_2_ to T_9_). For the non-inoculated control (i.e., T_1_), the roots of the seedlings were immersed only in the phosphate buffer. After a 2-hour incubation, one seedling was transplanted in each pot (four pots for each treatment) and the pots were kept in an open-field condition. When required, the pots were transferred in the rain shelter. The pot was watered with sterile deionized water to field capacity [when water was leached through bottom holes of the pots, this was considered maximum field capacity (Chandra et al., 2018; Jochum et al., 2019)] every day up to 45 days post planting (DPP). In order to impose water stress, the soil in the root region of each plant was carefully loosened. On 46 DPP, 50 mL bacterial suspension (suspended in phosphate buffer, pH 7.0) containing 10^8^ CFU mL^–1^ bacterial cells was applied to the rhizosphere region of each plant (T_2_ to T_9_ treatments), while to the control (T_1_), only 50 mL phosphate buffer was applied. After treatment, the watering in the pot was immediately stopped. Water stress was continued up to the 12th day (58 DPP).

##### Evaluation of plant growth and biochemical parameters

Plant height, number of primary branches, number of leaves, maximum leaf length, and maximum leaf width were recorded on the 12th day (58 DPP) of water stress. For biochemical parameters, the leaves were collected on this day. Then, each plant was carefully uprooted. The shoots and roots were separated, oven dried at 70°C for 72 h, and then weighed. To quantify the number of colonized bacteria in the rhizosphere (roots with soil), non-rhizosphere soil was discarded from the roots by hand shaking. Then, 1 g sample (roots with soil) was taken from the composite sample, homogenized, and serial diluted. Then, 100 μL diluted sample was spread on YEP agar plates (4 replications each). After a 48-hour incubation at 28°C, bacterial populations were counted.

##### Relative water content

Three fully expanded 3rd leaves from the top of the main stem were collected from each treatment. Five leaf discs (1 cm^2^) from each treatment were weighed. Then, the leaf discs were placed in distilled water and the turgid weight recorded after 24 h at 4°C. The discs were then oven-dried at 72°C until constant weights were attained, and the dry weight was determined. The relative water content (RWC) was calculated as follows:

$$RWC\left(\%\right)=\left[\frac{Freshweight-Dryweight}{Turgidweight-Dryweight}\right]\times 100$$

##### Chlorophylls and carotenoids

A UV-Vis spectrophotometer (Ultrospec 3000, Pharmacia Biotech, Cambridge, United Kingdom) was used to quantify chlorophyll (chl) *a*, chl *b*, total chl, and carotenoids from fresh leaf samples as described in Khan et al. (2017) with a few modifications. In brief, 50 mg fresh leaf sample was transferred into a glass test tube containing 5 mL 90% acetone, covered tightly, and kept in the dark at room temperature. After 48 h, the absorbance was measured at 663, 645, and 470, respectively. The result was expressed as mg g^–1^ fresh weight (FW). The formulae for computing chl *a*, chl *b*, total chl, and carotenoids were:

$$Chla=\left[12.7\left(A663\right)-2.69\left(A645\right)\right]\left[\frac{V}{1000\times W}\right]$$

$$Chlb=\left[22.9\left(A645\right)-4.68\left(A663\right)\right]\left[\frac{V}{1000\times W}\right]$$

$$TotalChl=\left(20.21\times A645+8.02\times A663\right)\left[\frac{V}{1000\times W}\right]$$

$$Caroteniods=\frac{\left[1000\left(A470\right)-2.270\times Chla-81.4\times Chlb\right]}{227}$$

where A (663, 645, 470) represents the optical density of the chlorophyll extract at the wave length of 663 nm, 645 nm, and 470 nm; V is the final volume (mL) of 90% acetone with chlorophyll extract; and W is the weight of the fresh leaf sample in g.

##### Electrolyte leakage

Electrolyte leakage (EL) was determined as described in Lutts et al. (1996) with a few modifications. In brief, 1-cm^2^ leaf discs (5 discs/treatment) were transferred into glass test tubes containing 10 mL distilled water. Then, electrical conductivity (EC) was recorded at room temperature. The sample-containing test tubes were kept in a water bath at 40°C for 30 min, and EC was measured. Afterward, the samples were boiled at 100°C, and EC was recorded after 30 min. Finally, EL was calculated using the following formula:

EL(%)=[ECat 40C∘-ECatroomtemperatureECat 100C∘]×100

#### Measurement of Lipid Peroxidation (MDA Level)

The MDA level was quantified as described in Vemanna et al. (2017) with a few modifications. In brief, 0.15 g leaf sample was homogenized in 2.5 mL of 5% (w/v) trichloroacetic acid (TCA) and 0.25% of thiobarbituric acid (TBA) and then centrifuged at 15000 rpm at 4°C for 10 min. The supernatant was mixed with an equal volume of TBA (0.5% in 20% TCA) and then boiled for 20 min at 100°C. The reaction was stopped by incubation on ice. The absorbance of the supernatant was measured at 532 and 600 nm, respectively. Nonspecific turbidity was corrected by subtracting the absorbance at 600 nm. The MDA content was calculated using its molar extinction co-efficient of 155 mM^–1^cm^–1^ and expressed as μmol g^–1^ FW.

### Proline Content

The proline content was estimated as described in Bates et al. (1973) with a few modifications. In brief, 0.1 g leaf was homogenized in 10 mL 3% aqueous sulfosalicylic acid (Sigma-Aldrich, St. Louis, MO, United States) and centrifuged at 15000 rpm for 15 min. Then, 2 mL acid-ninhydrin and 2 mL glacial acetic acid were mixed with 2 mL supernatant and cooked in a water bath at 100°C. After 30 min, the test tubes were transferred on ice to stop the reaction. Then, 4 mL toluene (Wako Pure Chemicals, Japan) was added. After a 10-min incubation at room temperature, the reaction mixture was vigorously mixed and the absorbance of the upper layer of the mixture containing toluene was measured at 520 nm using an UV-Vis spectrophotometer. The amount of proline was quantified from the standard curve using the following equation:

$$\begin{array}{c} Proline\left(\mu gpergfreshweight\right)\\ \;=\frac{\begin{array}{c} (\mu gpermLProline\times VolumeofToluene\\ \times VolumeofSulfosalicylicacid) \end{array}}{0.1\times 115.5} \end{array}$$

where 0.1 is the sample weight (g) and 115.5 is the molecular weight of proline.

#### Catalase Activity

In order to determine the catalase activity, 0.2 g leaf sample was homogenized in phosphate buffer (100 mM; pH 7.2) and then centrifuged at 14000 rpm for 15 min at 4°C. The supernatant (0.1 mL) containing the enzyme extract was added to a 1-mL reaction mixture in a glass test tube consisting of 100 mM phosphate buffer (pH 7.2), 0.1 μM EDTA, and 0.1% H_2_O_2_. Then, the decrease of H_2_O_2_ was determined by measuring the absorbance at 240 nm with a spectrophotometer and the catalase activity quantified using the following equation:

$$\begin{array}{c} Catalaseactivity\left(Upermgprotein\right)\\ =\frac{\left[\left(Absorbanceofblank-Absorbanceoftestedsample\right)\times 271\right]}{\frac{\left(60\times Volumeofenzymeextract\right)}{Proteinconcentrationinenzymeextract}} \end{array}$$

### Statistical Analysis

All the assays were performed in CRD with at least three replications and repeated at least twice unless otherwise stated. However, the effect of water-deficit stress on plant growth and the expression of the biochemical parameters were conducted only once. For the different parameters collected from the bacterial treatments, a one-way ANOVA test was done. The distribution of the data sets was normal and confirmed by the Shapiro–Wilk test. Heteroscedasticity of the data sets was checked by the Bartlett’s test and found homogenous/homoscedastic. The graphical distribution of datasets was analyzed by Q–Q plot. ANOVA, distribution of data, homogeneity of variance, and mean comparison of treatment effects were analyzed using the R software version 3.3.6. The Fisher’s least significant difference test was applied to compare the means.

## Results

### Isolation of Biofilm-Producing Rhizobacterial Strains

**FIGURE 1:**
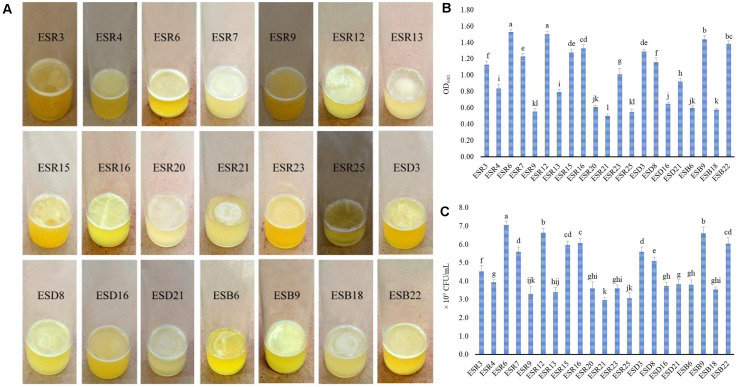
Production of AL biofilms by different rhizobacterial strains after a 72-hour incubation at 28°C in stationary conditions **(A)**. Biomass of AL biofilms determined at 600 nm **(B)**. Number of bacteria-coupled with AL biofilms **(C)**. The values are mean, and error bars indicate standard deviation (±) of the three independent experiments. Values having different letters are significantly different from each other according to Fisher’s least significant difference (LSD) test (*P* ≤ 0.001).

Based on colony morphology, 78 (seventy-eight) rhizobacterial strains (26 strains from each area) were isolated (data not shown). All these strains were tested for their ability to form AL and/or SAL biofilms on the glass test tubes containing SOBG broth at 28°C under static conditions. Only 21 (26.92%) strains produced delicate to stout AL biofilms after 72 h of incubation (Figure 1A). The remaining strains formed neither AL nor SAL biofilms even after 7 days of incubation (data not shown). In Rajshahi, 50% of the analyzed strains (ESR3, ESR4, ESR6, ESR7, ESR9, ESR12, ESR13, ESR15, ESR16, ESR20, ESR21, ESR23, and ESR25) produced biofilms, while only 15.38% strains forming the biofilms were isolated from Dinajpur (ESD3, ESD8, ESD16, and ESD21) and from Bogura (ESB6, ESB9, ESB18, and ESB22). All these biofilm-producing strains produced rough-surfaced biofilms (Figure 1A). Among these strains, ESR6, ESR12, and ESB9 built very thick and rigid biofilms, and the associated bacterial cells were not dispersed when the aggregates were agitated. Conversely, ESR9, ESR20, ESD16, and ESB18 produced very thin and fragile biofilms, and the cells of these biofilms were easily dispersed when disturbed. Furthermore, those biofilms generated by ESR7, ESR16, ESR23, ESD3, ESD8, and ESB22 were denser and stronger than the biofilms constructed by ESR9, ESR20, ESD16, or ESB18. Interestingly, the biofilms formed by ESR3, ESR13, ESR15, ESR21, ESR25, and ESB6 did not completely cover the surface of the standing culture. Thus, the biofilm characteristics, including thickness, strength, and covering the surface depended on the bacterial strains.

### Quantification of Biomass Biofilms and Bacteria Associated With Biofilms

The biomass biofilms (Figure 1B) and the bacterial counts in the biofilm matrices (Figure 1C) varied significantly (*P* ≤ 0.001) between the biofilm-producing strains. The highest amount of biomass biofilm was produced by ESR6 (OD_600_: 1.53), which was statistically similar to ESR12 (OD_600_: 1.50). ESB9 produced a biomass biofilm of OD_600_: 1.44), followed by ESB22 (OD_600_: 1.39) and ESR16 (OD_600_: 1.33). The lowest quantity of biomass biofilm was built by ESR21 with an OD_600_ at 0.50. However, the biomass biofilms differed not considerably between ESR3, ESD8, ESR4, and ESR13. Considering the bacterial counts (Figure 1C), the maximum CFU was noted in ESR6 (7.1 × 10^9^). The second highest CFU was recorded in both ESR12 and ESB9 (6.6 × 10^9^ each) followed by ESR16 (6.1 × 10^9^), ESR15 (6.0 × 10^9^), and ESB22 (6.0 × 10^9^). The minimum CFU was counted in ESR21 (3.0 × 10^9^). However, the CFU differed not remarkably between ESB2, ESB6, ESD16, ESR20, ESR25, and ESB18. Thus, also the quantity of biofilms and the bacterial numbers in these biofilms are influenced by the bacterial strains.

### Identification of Biofilm-Producing Rhizobacteria

**FIGURE 2:**
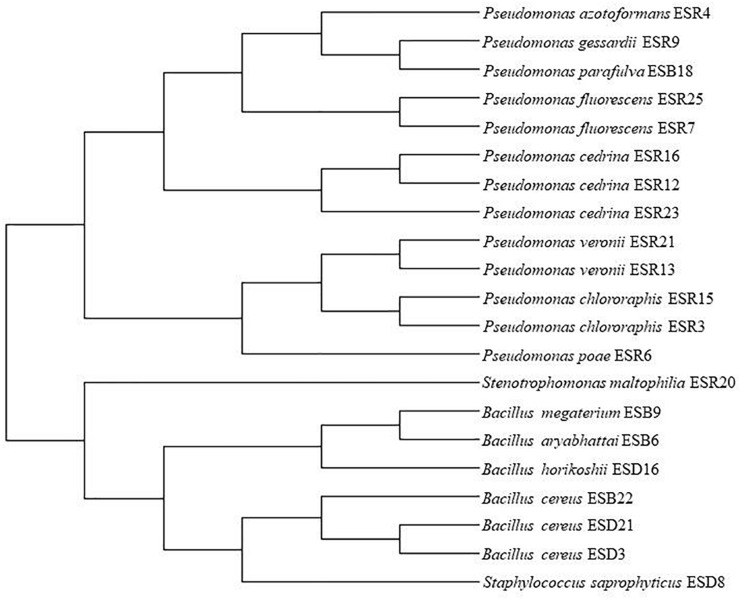
Phylogenetic tree. MUSCLE alignment and maximum likelihood (PhyML) method were used for tree generation with gBlock used for alignment refinement.

All the biofilm-producing rhizobacterial strains were identified based on 16S rRNA gene sequencing (Table 1). The strains ESR3 and ESR15 were identified as *Pseudomonas chlororaphis*, ESR7 and ESR25 as *P. fluorescens*, ESR13 and ESR21 as *P. veronii*, and ESR12, ESR16, and ESR23 as *P. cedrina*. The strains ESR4, ESR6, ESR9, and ESR20 belonged to *P*. *azotoformans*, *P. poae*, *P. gessardii*, and *Stenotrophomonas maltophilia*, respectively. ESD3, ESD21, and ESB22 were recognized as *Bacillus cereus*, and ESD8 and ESD16 as *Staphylococcus saprophyticus* and *B. horikoshii*, respectively. ESB6, ESB9, and ESB18 were identified as *B. aryabhattai*, *B. megaterium*, and *P. parafulva*. The sequence data were submitted to the NCBI GenBank, and the allocated accession number is shown in Table 1. A phylogenetic tree was also constructed using the 16S rRNA gene sequence data (Figure 2).

### FTIR Spectroscopy

**FIGURE 3:**
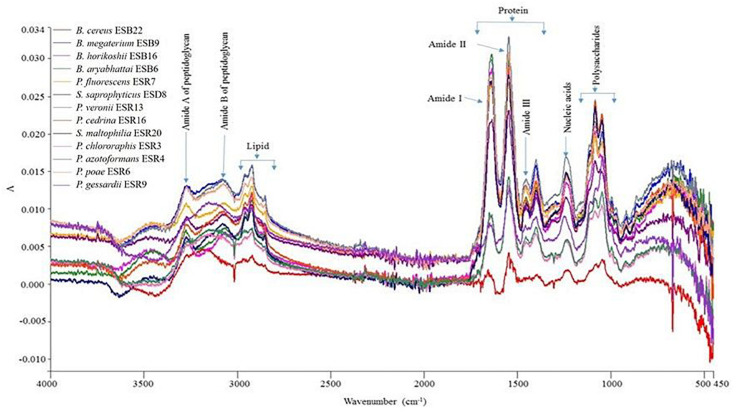
Detection of organic macromolecules constructed by different biofilm-producing PGPR by FTIR.

The matrix components of the biofilms produced by the identified rhizobacterial strains used in this study were not yet characterized. In the present study, we characterized the biofilm matrix formed by these bacterial strains by the FTIR spectroscopy (Figure 3). All the matrices were dominated by protein compounds, producing peaks at amide I (1600–1700 cm^–1^), amide II (1500–1600 cm^–1^), and amide III (1240–1350 cm^–1^) regions. Also, these bacterial strains generated a substantial amount of polysaccharides which produced intense peaks near 900–1150 cm^–1^. Moreover, the peaks within the 1220–1250-cm^–1^ band region demonstrate the presence of nucleic acids in the biofilm matrices (Naumann, 2001). Nevertheless, the 2800–2970-cm^–1^, 3200-cm^–1^, and 2955-cm^–1^ domains also indicate the presence of lipids (Mosharaf et al., 2018), amide A of peptidoglycan (Naumann et al., 1982), and amide B of peptidoglycan (Naumann, 2001), respectively. Thus, proteins, polysaccharides, nucleic acids, lipids, and amide A- and amide B of peptidoglycan are all components of the biofilm matrices for these bacteria.

### Congo Red and Calcofluor Binding Assays

**FIGURE 4:**
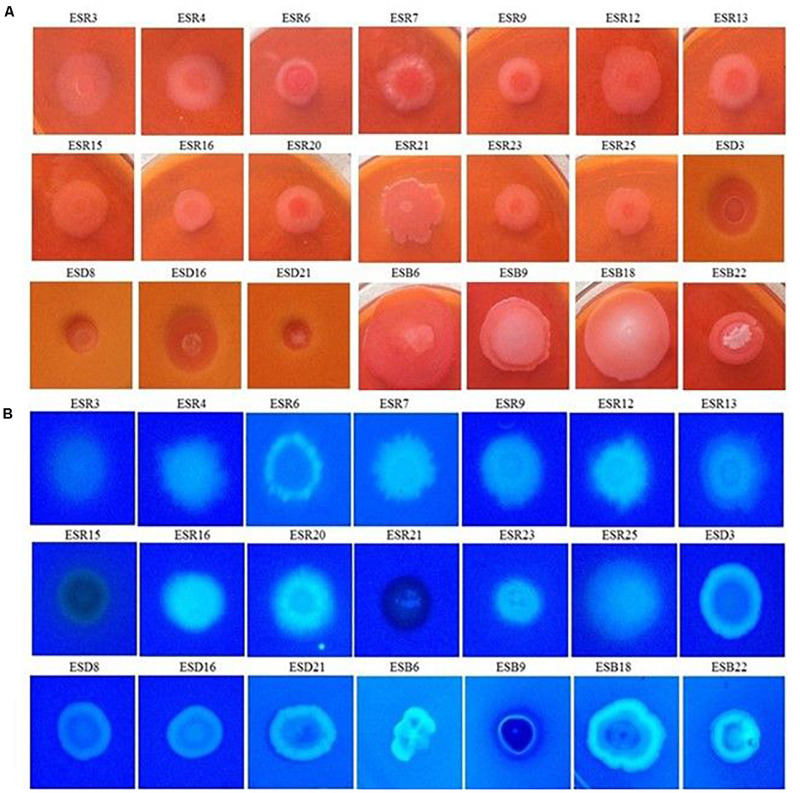
Binding assays. Different rhizobacterial strains were spotted onto Congo red (40 μg mL^–1^) **(A)**, and Calcofluor (200 μg mL^–1^) **(B)** agar plates were then incubated at 28°C in static conditions for 48 h. Photographs represent one of three experiments, which gave similar results.

All these bacterial strains were tested for their abilities to bind Congo red. Indeed, all these strains were able to bind Congo red and developed the typical red, dry, and rough (rdar) phenotype on Congo red agar plates (Figure 4A). The results suggested that they produced both curli fimbriae and cellulose (Römling, 2005; Milanov et al., 2015). On Calcofluor agar plates, *P. fluorescens* ESR7, *P. cedrina* (ESR12, ESR16, and ESR23), *S. maltophilia* ESR20, *B. cereus* (ESD3, ESD21, and ESB22), and *B. aryabhattai* ESB6 and *P. parfulva* ESB18 exhibited a strong fluorescence (Figure 4B), while the remaining bacterial strains only showed a weak or moderate fluorescence (Figure 4B). Hence, all these strains expressed cellulose (Zogaj et al., 2001; Römling, 2005). However, the quantity of cellulose production might vary between these bacterial strains.

### SEM Analysis

**FIGURE 5:**
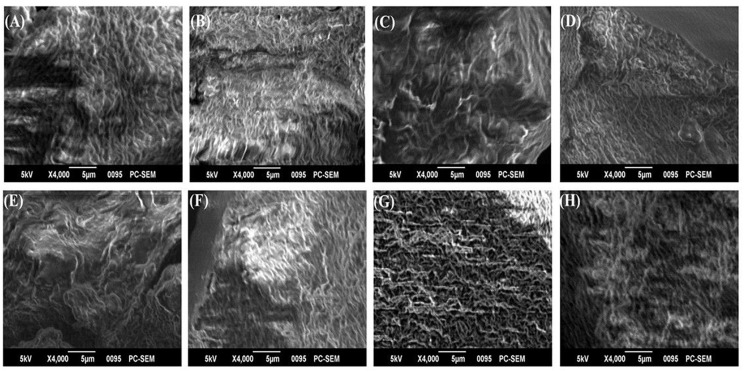
SEM images of biofilm matrices generated by *P. chlororaphis* ESR3 **(A)**, *P. azotoformans* ESR4 **(B)**, *P. poae* ESR6 **(C)**, *P. gessardii* ESR9 **(D)**, *P. cedrina* ESR12 **(E)**, *P. veronii* ESR13 **(F)**, *S. maltophilia* ESR20 **(G)**, and *B. megaterium* ESB9 **(H)**.

The biofilm matrices of *P. chlororaphis* ESR3, *P. azotoformans* ESR4, *P. poae* ESR6, *P. gessardii* ESR9, *P. cedrina* ESR12, *P. veronii* ESR13, *S. maltophilia* ESR20, and *B. megaterium* ESB9 were interlinked, compact, and highly fibrous (Figure 5). Numerous ribbon-like fibers were also observed. These ribbon-like fibers are known as nanocellulose fibers (Jahn et al., 2011; Hu et al., 2013). Thus, these bacterial strains produced nanocellulose-rich polysaccharides.

### Production of IAA

The qualitative assessment of IAA using Salkowski’s reagent showed that except for *B. cereus* ESD3 and *S. saprophyticus* ESD8, all the analyzed rhizobacterial strains exhibited the color change ranging from light pink to dark red, suggesting the positivity for production of auxin-related compounds (Glickmann and Dessaux, 1995) including IAA (Supplementary Figure S1). When quantified using a spectrophotometer, the IAA production ranged from 4.45 to 59.57 μg mL^–1^ (Table 2). The highest amount of IAA was synthesized by *B. horikoshii* ESD16 (59.57 μg mL^–1^) followed by *P. poae* ESR6 (31.17 μg mL^–1^). The least amount of IAA was produced by *B. cereus* ESB22 (4.45 μg mL^–1^).

**FIGURE 6:**
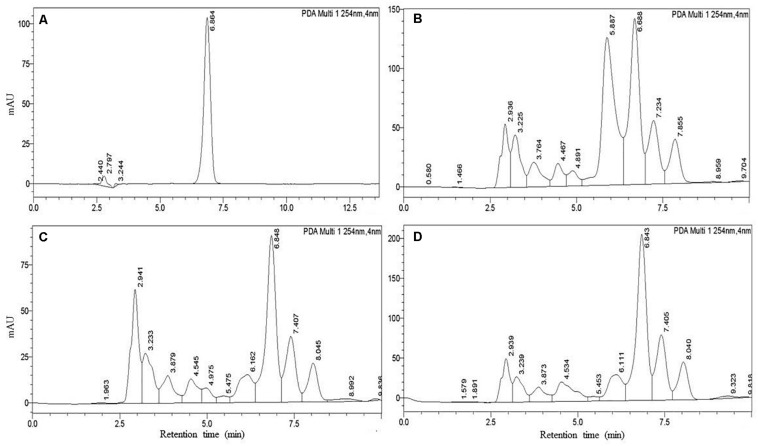
Detection of IAA using HPLC. **(A)** IAA standard (50 μg mL^–1^), **(B)** *P. cedrina* ESR12, **(C)** *P. chlororaphis* ESR15, and **(D)** *B. aryabhattai* ESB6.

We also confirmed IAA production quantitatively by the HPLC system. The results showed that standard IAA (Duchefa, Netherland) elution in HPLC developed a major peak at a retention time of 6.864 min, while IAA extracted from the isolates developed a sharp peak at a retentions time ranging from 6.68 to 6.82 min. We found that the detection sensitivity of IAA by HPLC is 2- to 3-fold higher than spectrometric quantification. In HPLC, we quantified IAA ranging from 19.3 to 71.7 μg mL^–1^ (Table 2). The isolate *B. aryabhattai* ESB6 and *P. cedrina* ESR16 synthesized the highest and lowest amount of IAA, respectively, as the spectrometric analysis IAA was not detected from the isolates *B. cereus* ESD3 and *S. saprophyticus* ESD8. The representative chromatogram of standard IAA (50 μg mL^–1^) and *P. cedrina* ESR12, *P. chlororaphis* ESR15, and *B. aryabhattai* ESB6-extracted IAA is presented in Figure 6.

**TABLE 2:** IAA production and nutrient acquisition by different biofilm-producing rhizobacteria.

	IAA production (μg/mL)		Nitrogen fixation		Phosphate solubilization index			
Strains	Spectrophotometric assay	HPLC	Growth on N_2_ free medium	Expression of *nifH*	Tricalcium phosphate	Rock phosphate	K solubilization	Zn solubilization index
ESR3	22.03 ± 1.70 de	43.20 ± 10.02 cd	+	+	6.43 ± 0.11 a	2.50 ± 0.24 fg	+	2.65 ± 0.29 b
ESR4	22.85 ± 2.22 de	31.23 ± 7.77 defg	+	+	2.79 ± 0.04 ef	2.83 ± 0.24 efg	+	3.42 ± 0.59 ab
ESR6	31.17 ± 2.20 b	59.52 ± 9.45 ab	+	+	5.57 ± 0.21 b	4.23 ± 0.80 ab	–	3.17 ± 1.18 ab
ESR7	18.54 ± 1.57 fg	24.58 ± 8.54 fg	+	+	2.40 ± 0.02 h	4.08 ± 0.12 abc	+	2.94 ± 0.62 ab
ESR9	20.69 ± 1.74 ef	48.83 ± 10.07 bc	+	+	2.90 ± 0.07 e	4.19 ± 0.67 ab	+	3.44 ± 0.09 ab
ESR12	23.88 ± 2.33 d	67.14 ± 4.04 a	+	+	2.69 ± 0.11 fg	3.24 ± 0.13 cdef	–	3.29 ± 0.20 ab
ESR13	22.78 ± 1.05 de	33.13 ± 7.64 defg	+	+	6.51 ± 0.04 a	4.50 ± 0.24 a	–	2.86 ± 0.37 ab
ESR15	18.25 ± 1.40 fg	38.78 ± 5.20 cde	+	+	4.46 ± 0.12 c	4.67 ± 0.0 a	+	2.40 ± 0.14 b
ESR16	22.80 ± 1.09 de	19.30 ± 8.02 g	+	+	2.85 ± 0.06 ef	4.21 ± 0.65 ab	+	3.06 ± 0.09 ab
ESR20	21.67 ± 0.79 de	44.50 ± 10.69 cd	+	+	2.56 ± 0.02 gh	3.04 ± 0.41 def	–	2.91 ± 0.48 ab
ESR21	20.43 ± 0.70 ef	33.19 ± 8.62 def	+	+	3.69 ± 0.36 d	3.50 ± 0.24 bcde	–	2.30 ± 0.14 b
ESR23	26.55 ± 1.23 c	51.47 ± 11.53 bc	+	+	6.47 ± 0.35 a	3.83 ± 0.24 abcd	–	3.20 ± 1.13 ab
ESR25	14.32 ± 0.90 hi	28.22 ± 11.02 efg	+	+	2.79 ± 0.07 ef	2.17 ± 0.24 g	–	3.88 ± 0.18 a
ESD3	–		+	+	–	4.67 ± 0.47 a	+	–
ESD8	–		+	+	2.85 ± 0.06 ef	4.08 ± 0.59 abc	+	–
ESD16	59.57 ± 3.80 a	21.52 ± 4.93 fg	+	+	2.14 ± 0.11 j	4.67 ± 0.47 a	+	–
ESD21	13.59 ± 0.73 i	31.45 ± 7.08 defg	+	+	2.18 ± 0.01 ij	2.00 ± 0.35 g	–	–
ESB6	21.73 ± 0.89 de	71.70 ± 7.00 a	+	+	2.38 ± 0.01 hi	2.50 ± 0.24 fg	–	–
ESB9	16.16 ± 1.29 gh	25.64 ± 7.94 efg	+	+	2.11 ± 0.06 j	–	–	–
ESB18	4.83 ± 0.90 j	20.08 ± 7.51 fg	+	+	–	–	–	–
ESB22	4.55 ± 0.19 j	23.50 ± 8.02 fg	+	+	–	–	–	–

*+ = Positive, – = Negative. The values are mean of the three independent experiments. ± indicate standard deviation. Values having different letters are significantly different from each other according to Fisher’s least significant difference (LSD) test (P ≤ 0.001).*

### Fixation and Solubilization of Nutrients

All the bacterial strains formed colonies on N-free solid LG agar plates (Table 2). Genomic DNAs of these bacterial strains were also amplified for the *nifH* gene by PCR. The primer pairs of Ueda19F and Ueda19R and KAD3-F and DVV-R were amplified at 389 and 310 bp, respectively (Table 2 and Supplementary Figure S2). Thus, all these rhizobacterial strains have nitrogen fixation activity.

All the rhizobacterial strains were evaluated for their abilities to solubilize P from two sources including tricalcium phosphate and rock phosphate. Except *P. parafulva* ESB18 and *B. cereus* (ESD3 and ESB22), all other strains formed a clear halo zone around the colonies on NBRIP agar containing 0.5% tricalcium phosphate (data not shown). All the rhizobacterial strains also developed a clear halo zone around the colonies on NBRIP containing 0.8% rock phosphate except *B. megaterium* ESB9, *P. parafulva* ESB18 and *B. cereus* ESB22 (data not shown). Thus, most of the rhizobacterial strains are P solubilizers. The PSI value of tricalcium phosphate and rock phosphate was calculated, fluctuating from 2.11 to 6.51 and 2.00 to 4.67, respectively (Table 2). In the case of tricalcium phosphate, *P. veronii* ESR13 exhibited the highest PSI value (6.51) which was statistically similar with *P. chlororaphis* ESR3 (6.43) and *P. cedrina* ESR23 (6.47). Conversely, the lowest PSI value (2.11) was noted in *B. megaterium* ESB9. In the case of rock phosphate, the maximum PSI value (4.67) is displayed in *P. chlororaphis* ESR15, *B. cereus* ESD3, and *B. horikoshii* ESD21 which did not significantly differ with that of *P. poae* ESR6 (4.23), *P. fluorescens* ESR7 (4.08), *P. gessardii* ESR7 (4.19), *P. veronii* ESR13 (4.50), *P. cedrina* ESR16 (4.21), and *S. saprophyticus* (4.08). Furthermore, when the P-solubilizing strains were inoculated in NBRIP broth supplemented with 0.5% tricalcium phosphate or 0.8% rock phosphate at pH 7.0 and incubated at 28°C for 96 h under shaking conditions, the pH value of this broth decreased from 7.0 to a pH of 4.5–5.5 (data not shown). This result indicated that these rhizobacterial strains secreted organic acids to solubilize tricalcium phosphate and rock phosphate (Ahemad and Kibret, 2014).

Only *P. chlororaphis* (ESR3 and ESR15), *P. azotoformans* ESR4, *P. fluorescens* ESR7, *P. gessardii* ESR9, *P. cedrina* ESR16, *B. cereus* ESD3, *S. saprophyticus* ESD8, and *B. horikoshii* ESD16 were able to form a clear halo zone around their colonies spotted onto Aleksandrov agar plates containing potassium aluminum silicate (Table 2), suggesting that they are K solubilizers.

None of the bacterial strains solubilized with Zn when ZnO or ZnCO_3_ was used as the Zn source (data not shown). Interestingly, when Zn_3_(PO_4_)_2_ was provided as a Zn source, a clear halo zone (data not shown) was observed around the colonies of *P. chlororaphis* (ESR3 and ESR15), *P. azotoformans* ESR4, *P. poae* ESR6, *P. fluorescens* (ESR7 and ESR25), *P. gessardii* ESR9, *P. cedrina* (ESR12, ESR16, and ESR23), *P. veronii* (ESR13, ESR21), and *S. maltophilia* ESR20, indicating that they are in fact Zn solubilizers. In this experiment, the ZnSI value ranged from 2.30 to 3.88 (Table 2). Among the Zn solubilizers, *P. fluorescens* ESR25 showed a maximum value of 3.88 though it was not significantly (*P* ≤ 0.001) different to the ZnSI values of *P. azotoformans* ESR4, *P. poae* ESR6, *P. fluorescens* ESR7, *P. gessardii* ESR9, *P cedrina* (ESR12, ESR16 and ESR23), and *P. veronii* ESR13.

### Expression of Siderophores

**FIGURE 7:**
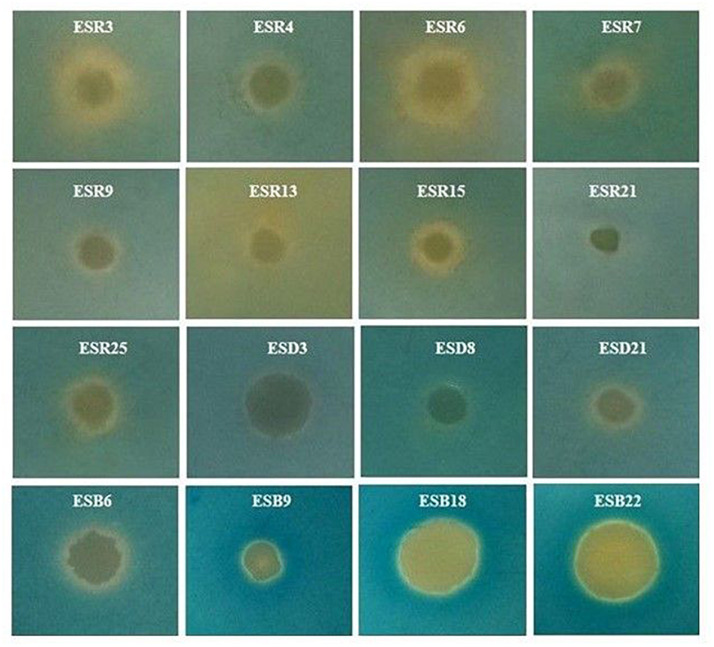
Expression of siderophores. Each bacterium (2 μL, ca. 10^7^ CFU mL^–1^) was spotted onto the center of the LB agar plates and incubated at 28°C for 20 h. Then, 10 mL O-CAS broth was added over those LB agar plates and kept at 28°C in static conditions for 10 h. Photographs represent one of three experiments, which gave similar results.

Developing varying colors on the O-CAS agar medium is linked with the expression of different types of siderophores: a purple color is associated with catechol-type siderophores, a light orange/orange color is related with hydroxamate-type siderophores, a light yellow color is connected with carboxylate-type siderophores, and a yellow color is linked with both hydroxamate- and carboxylate-type siderophores (Pérez-Miranda et al., 2007). In this study, *P cedrina* (ESR12, ESR16, and ESR23), *S. maltophilia* ESR20, and *B. horikoshii* ESD16 were incapable of changing colors (data not shown). In contrast *P. chlororaphis* (ESR3 and ESR15), *P. azotoformans* ESR4, *P. poae* ESR6, *P. fluorescens* (ESR7 and ESR25), *P. gessardii* ESR9, *P. veronii* ESR13, and *B. cereus* ESD21 produced a light orange color (Figure 7), suggesting that these bacterial strains produced hydroxamate-type siderophores. *B. aryabhattai* ESB6, *B. megaterium* ESB9, *B. cereus* ESB22, and *P. parafulva* ESB18 developed a yellow color, indicating that they generated both hydroxamate- and carboxylate-type siderophores. *P. veronii* ESR21, *B. cereus* ESD3, and *S. saprophyticus* ESD8 induced a light yellow color, specifying that they constructed carboxylate-type siderophores (Figure 7).

### Production of Volatile Compounds

Volatile compounds produced by the different rhizobacterial strains are shown in Table 3. Only *B. horikoshii* ESD16 and *B. megaterium* ESB9 produced acetoin. On the other hand, *P. poae* ESR6, *P. cedrina* (ESR12 and ESR16), *S. maltophilia* ESR20, *P. veronii* ESR21, *B. cereus* (ESD3 and ESB22), *B. aryabhattai* ESB6, *B. megaterium* ESB9, and *P. parfulva* ESB18 synthesized indole. A total of 95.23% strains produced ammonia (Table 3 and Supplementary Figure S3). Based on color intensity, *P. chlororaphis* (ESR3 and ESR15), *P. azotoformans* ESR4, *P. poae* ESR6, *P. fluorescens* ESR7, *P. gessardii* ESR9, *P. cedrina* (ESR12, ESR16, and ESR23), *B. cereus* (ESD21 and ESB22), *B. aryabhattai* ESB6, and *B. megaterium* ESB9 were strong ammonia producers. However, only *P. chlororaphis* (ESR3 and ESR15) and *S. saprophyticus* ESD8 produced HCN (Table 3 and Supplementary Figure S4).

**TABLE 3:** Production of volatile compounds and hydrolytic enzymes by different biofilm-producing rhizobacterial strains.

	Volatile compounds				Hydrolytic enzymes							
Strains	Acetoin	Indole	NH_3_	HCN	ACC deaminase	Catalase	Oxidase	Gelatinase	Arginine dihydrolase	Lipase	Cellulase	Protease
ESR3	–	–	+++^*c*^	+^*a*^	+	+	+	+	+	+	+	–
ESR4	–	–	+++^*c*^	–	+	+	+	+	+	+	+	–
ESR6	–	+	+++^*c*^	–	+	+	+	+	+	–	+	–
ESR7	–	–	+++^*c*^	–	+	+	+	+	+	+	+	–
ESR9	–	–	+++^*c*^	–	+	+	+	+	+	+	+	–
ESR12	–	+	+++^*c*^	–	+	+	+	+	+	+	+	–
ESR13	–	–	++^*b*^	–	+	+	+	+	+	+	+	–
ESR15	–	–	+++^*c*^	+++^*c*^	+	+	+	+	+	+	+	–
ESR16	–	+	+++^*c*^	–	+	+	+	+	+	+	+	–
ESR20	–	+	–	–	+	+	+	+	-	+	+	+
ESR21	–	+	+^*a*^	–	+	+	+	–	+	–	+	+
ESR23	–	–	+++^*c*^	–	+	+	+	+	+	+	+	+
ESR25	–	–	+^*a*^	–	+	+	+	–	+	+	+	–
ESD3	–	+	++^*b*^	–	+	+	+	+	+	–	+	+
ESD8	–	–	+^*a*^	+^*a*^	+	+	–	+	+	+	+	+
ESD16	+	–	+^*a*^	–	+	+	–	+	–	+	+	+
ESD21	–	–	+++^*c*^	–	+	+	–	+	+	+	+	+
ESB6	–	+	+++^*c*^	–	+	+	–	+	–	+	+	–
ESB9	+	+	+++^*c*^	–	+	+	–	+	–	+	+	+
ESB18	–	+	+^*a*^	–	+	+	+	–	+	+	+	+
ESB22	–	+	+++^*c*^	–	+	+	+	–	–	+	+	+

*+ = Positive, – = Negative, +^a^ = Weak, ++^b^ = Moderate, and +++^c^ = Strong.*

### Production of Hydrolytic Enzymes

The hydrolytic enzyme production in the different rhizobacteria is shown in Table 3. All these bacterial strains were positive for ACC deaminases, catalases, and cellulases. On the other hand, *S. saprophyticus* ESD8, *B. horikoshii* ESD16, *B. cereus* ESD21, *B. aryabhattai* ESB6, and *B. megaterium* ESB9 were negative for oxidases. Most of these strains (80.95%) were positive for gelatinase whereas *P. veronii* ESR21, *P. fluorescens* ESR25, *P. parfulva* ESB18, and *B. cereus* ESB22 were gelatinase negative. A good number of bacterial strains (76.19%) were also positive for arginine dihydrolase. Except *P. poae* ESR6, *P. veronii* ESR21, and *B. cereus* ESD3, all other bacterial strains showed a lipase production. *S. maltophilia* ESR20, *P. veronii* ESR21, *P. cedrina* ESR23, *B. cereus* (ESD3, ESD21, and ESB22), *S. saprophyticus* ESD8, *B. horikoshii* ESD16, *B. megaterium* ESB9, and *P. parafulva* ESB18 also produced proteases.

### Biocontrol of Phytopathogenic Bacteria *in vitro*

**FIGURE 8:**
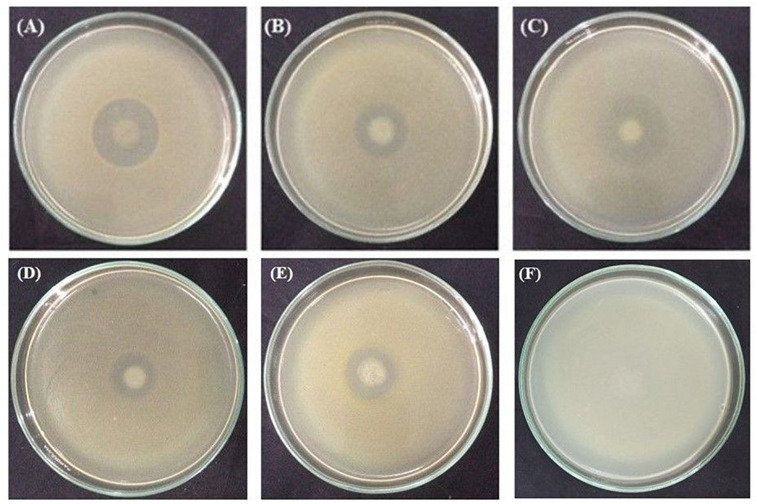
Antagonism tests. Inhibition of *X. campestris* pv. *campestris* ATCC 33913 by *P. cedrina* ESR12 **(A)**, *P. chlororaphis* ESR15 **(B)** and *B. cereus* ESD3**(C)**. Inhibition of *R. solanacearum* ATCC^®^ 11696^TM^ by *P. chlororaphis* ESR15 **(D)**, *B. cereus* ESD21 **(E)**, and negative control **(F)**. Photographs represent one of three experiments, which gave similar results.

All these rhizobacterial strains were tested for their antagonistic activities against three in tomato catastrophic phytopathogenic bacteria *X. campestris* pv. *campestris* ATCC 33913, *R. solanacearum* ATCC^®^ 11696^TM^, and *P. carotovorum* subsp. *carotovorum* PCC8 (Figure 8). None of the rhizobacterial strains were able to restrict the growth of *P. carotovorum* subsp. *carotovorum* PCC8 (data not shown). Interestingly, the growth of *X. campestris* pv. *campestris* ATCC 33913 was inhibited by *P. cedrina* ESR12, *P. chlororaphis* ESR15, and *B. cereus* ESD3, *P. chlororaphis* ESR15, and *B. cereus* ESD21, on the other hand, controlled the growth of *R. solanacearum* ATCC^®^ 11696^TM^.

### Abiotic Stress Tolerance

The abiotic stress tolerance performance of different rhizobacteria is depicted in Table 4. Except for *P. parafulva* ESB18, all other rhizobacterial strains were drought tolerant. All the bacterial strains grew at 37°C. *P. cedrina* ESR16 and ESR23, *S. maltophilia* ESR20, *P. veronii* ESR21, *B. cereus* (ESD3, ESD21, and ESB22), *B. horikoshii* ESD16, *B. megaterium* ESB9, and *P. parafulva* ESB18 also grew at 42°C. Surprisingly, however, *B. megaterium* ESB9, *B. cereus* ESB22, and *P. parafulva* ESB18 were even able to grow at 50°C. In regard to pH tolerance, *P. chlororaphis* ESR3, *B. cereus* (ESD3 and ESD21), *S. saprophyticus* ESD8, *B. aryabhattai* ESB6, *B. megaterium* ESB9, and *P. parafulva* ESB18 were unable to grow at pH 4.0 in contrast to the other strains. All the bacterial strains grew at pH 9.0. Except for *P. veronii* (ESR13 and ESR21) and *P. fluorescens* ESR25, all other strains also grew at pH 10. The salt-tolerance test revealed that all the bacterial strains were able to grow at 5% NaCl except for *P. fluorescens* ESR25 and *S. saprophyticus* ESD8. *S. saprophyticus* ESD8, *B. horikoshii* ESD16, *B. cereus* (ESD3 and ESD21), *B. aryabhattai* ESB6, and *B. megaterium* ESB9 tolerated 10% NaCl, and only *B. cereus* ESD3 and *B. aryabhattai* ESB6 were able to grow at 15% NaCl. Thus, most of the biofilm-producing bacteria not only survived under drought conditions but also survived high temperatures, acidic to alkaline conditions, and high NaCl.

**TABLE 4:** Abiotic stress tolerance of different biofilm-producing rhizobacterial strains.

	Drought	Temperature (°C)			pH			Salinity (% NaCl)		
Strains	25% PEG 6000	37	42	50	4.0	9.0	10	5	10	15
ESR3	+	+	–	–	–	+	+	+	–	–
ESR4	+	+	–	–	+	+	+	+	–	–
ESR6	+	+	–	–	+	+	+	+	–	–
ESR7	+	+	–	–	+	+	+	+	–	–
ESR9	+	+	–	–	+	+	+	+	–	–
ESR12	+	+	–	–	+	+	+	+	–	–
ESR13	+	+	–	–	+	+	-	+	–	–
ESR15	+	+	–	–	+	+	+	+	–	–
ESR16	+	+	+	–	+	+	+	+	–	–
ESR20	+	+	+	–	+	+	+	+	–	–
ESR21	+	+	+	–	+	+	–	+	–	–
ESR23	+	+	+	–	+	+	+	+	–	–
ESR25	+	+	–	–	+	+	–	–	–	–
ESD3	+	+	+	–	–	+	+	+	+	+
ESD8	+	+	–	–	–	+	+	–	+	–
ESD16	+	+	+	–	+	+	+	+	+	–
ESD21	+	+	+	–	–	+	+	+	+	–
ESB6	+	+	–	–	–	+	+	+	+	+
ESB9	+	+	+	+	–	+	+	+	+	–
ESB18	–	+	+	+	–	+	+	+	–	–
ESB22	+	+	+	+	+	+	+	+	–	–

*+ = Positive; – = Negative.*

### Effect of Drought Stress on Biofilm Formation

Some selected biofilm-producing PGPR were further examined for their abilities to form biofilms on SOBG containing 25% PEG 6000 which mimics water-stress conditions (Supplementary Figure S5). Compared to PEG-deficient SOBG, biofilm formation was only slightly reduced in *P. azotoformans* ESR4 and *P. poae* ESR6. Interestingly, *P. gessardii* ESR9, *P. cedrina* ESR12, *P. chlororaphis* ESR15, *S. maltophilia* ESR20, and *B. aryabhattai* ESB6 produced even thicker biofilms on SOBG containing 25% PEG 6000 as compared to PEG-deficient SOBG. Importantly, most of the bacterial cells were attached to the biofilms in SOBG containing 25% PEG 6000, while the number of planktonic cells was higher in PEG-deficient SOBG. These results suggest that all the tested rhizobacteria might be able to form biofilms in plants under water-deficit conditions but the quantity might vary.

### *In vivo* Plant Growth Promotion Under Water Deficit-Stressed Conditions

**FIGURE 9:**
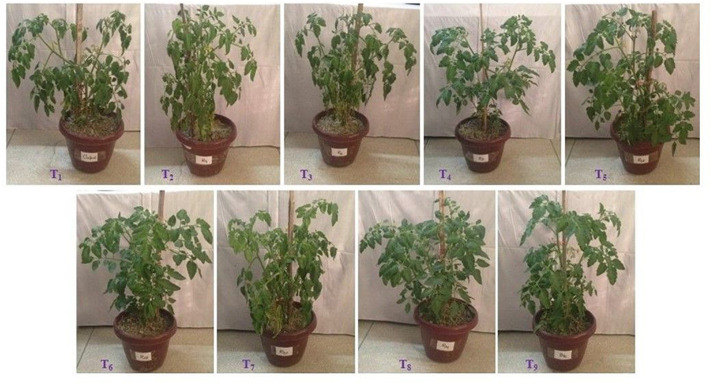
Effect of bacterial inoculation on growth of tomato plants under water deficit stressed conditions. Photographs were taken on 12th day of water stress. T_1_ = Without bacterial inoculation, T_2_, T_3_, T_4_, T_5_, T_6_, T_7_, T_8_, and T_9_ = Tomato plants inoculated with *P. azotoformans* ESR4, *P. poae* ESR6, *P. gessardii* ESR9, *P. cedrina* ESR12, *P. chlororaphis* ESR15, *S. maltophilia* ESR20, *P. veronii* ESR21, and *B. aryabhattai* ESB6, respectively.

We also analyzed the effect of biofilm-producing PGPR on tomato plants grown under water-deficit stress. The non-inoculated plants were wilted, and the leaves started curling on the 4th day (50 DPP) of water stress. This type of stress responses occurred on the 6th day of water stress when plants were inoculated with *P. azotoformans* ESR4 and *P. poae* ESR6 (data not shown). However, only the leaf-curling symptom but not the wilting one was observed in *P. gessardii* ESR9-, *P. cedrina* ESR12-, and *S. maltophilia*-treated plants on the 11th day (57 DPP) of water stress (Figure 9). Interestingly, *P. chlororaphis* ESR15- and *B. aryabhattai* ESB6-inoculated plants did not display any wilts and/or leaf-curling symptoms even after 12 days of water stress (Figure 9). This indicates a difference among the PGPR to mitigate water-deficit stress *in vivo* in field-grown tomato plants.

Data on plant growth-related traits are shown in Table 5. The highest increase in plant height (16.7%) was observed in *P. azotoformans* ESR4-inoculated plants, though this was statistically akin with *S. maltophilia* ESR20 and *B. aryabhattai* ESB6, showing 13.4 and 12.7% of height increase, respectively. Plant height was also improved by inoculation with *P. poae* ESR6, *P. gessardii* ESR9, *P. cedrina* ESR12, *P. chlororaphis* ESR15, and *P. veronii* ESR21, displaying a 10.7, 8.7, 8.1, 6.7, and 2.7% increase compared to non-inoculated plants, respectively.

**TABLE 5:** Growth of tomato plants and colonization of bacteria as influenced by inoculation of different biofilm-producing bacterial strains under water deficit-stressed conditions.

Treatments	Plant height (cm)***	Number of primary branches plant^–^^1^*	Number of leaves plant^–^^1^***	Maximum leaf length (cm)^α^	Maximum leaf width (cm)*	Dry matter production g plant^–^^1^		Bacterial CFU g^–^^1^ rhizosphere (roots along with soil)***
						Root***	Shoot**	
T_1_	49.67 ± 1.15 e	5.33 ± 0.58 c	15.33 ± 0.58 e	25.33 ± 2.08 b	17.67 ± 4.04 d	1.93 ± 0.01 f	13.26 ± 0.48 d	3.15E+5 ± 6.36E+4 d
T_2_	58.00 ± 3.00 a	6.67 ± 0.58 ab	23.00 ± 2.00 a	28.00 ± 1.00 ab	24.00 ± 3.00 abc	2.89 ± 0.04 a	17.26 ± 0.23 abc	4.33E+10 ± 2.47E+9 bc
T_3_	55.00 ± 1.00 bc	6.67 ± 1.15 ab	21.00 ± 2.00 ab	27.33 ± 2.31 ab	19.33 ± 1.53 cd	2.73 ± 0.04 b	18.02 ± 0.08 ab	4.18E+10 ± 1.06E+9 c
T_4_	54.00 ± 1.00 bc	5.33 ± 0.58 c	18.33 ± 0.58 bcd	26.33 ± 0.58 b	21.00 ± 0.02 cd	1.99 ± 0.04 e	15.30 ± 0.30 bcd	5.57E+10 ± 4.24E+8 a
T_5_	53.67 ± 1.53 bcd	5.67 ± 0.58 bc	19.33 ± 0.58 bc	26.33 ± 2.08 b	22.00 ± 2.00 abcd	2.46 ± 0.04 c	15.54 ± 0.20 bcd	4.5E+10 ± 7.07E+8 b
T_6_	53.00 ± 1.73 cd	5.67 ± 0.58 bc	17.67 ± 0.58 cde	27.67 ± 1.53 ab	23.67 ± 2.52 abc	2.34 ± 0.04 d	15.46 ± 0.02 bcd	4.37E+10 ± 2.33E+9 bc
T_7_	56.33 ± 1.53 ab	6.33 ± 0.58 abc	20.67 ± 3.06 ab	28.00 ± 3.61 ab	26.67 ± 5.51 a	1.98 ± 0.04 e	16.72 ± 0.20 abc	4.28E+10 ± 7.07E+8 bc
T_8_	51.00 ± 1.00 de	5.67 ± 0.58 bc	15.67 ± 1.15 de	27.33 ± 0.58 ab	21.67 ± 2.08 bcd	2.33 ± 0.04 d	14.19 ± 0.20 cd	4.45E+10 ± 4.94E+8 bc
T_9_	56.00 ± 2.00 ab	7.00 ± 0.02 a	22.50 ± 1.50 a	30.50 ± 1.50 a	26.50 ± 0.50 ab	2.35 ± 0.03 d	19.83 ± 5.99 a	4.2E+10 ± 1.41E+9 bc

T_1_ = Without bacterial inoculation, T_2_, T_3_, T_4_, T_5_, T_6_, T_7_, T_8_, and T_9_ = Tomato plants inoculated with P. azotoformans ESR4, P. poae ESR6, P. gessardii ESR9, P. cedrina ESR12, P. chlororaphis ESR15, S. maltophilia ESR20, P. veronii ESR21, and B. aryabhattai ESB6, respectively. Data are means ± standard deviations. Different letters in column imply significant difference among treatments. ***, **, *, and ^α^ indicate significant difference at 0.1% (P ≤ 0.001), 1% (P ≤ 0.01), 5% (P ≤ 0.05), and 10% (P ≤ 0.1), respectively.

Also, the number of primary branches provides an evidence for the health of the tomato plants. The highest number of primary branches (7.0 plant^–1^) was found in *B. aryabhattai* ESB6-inoculated plants followed by *P. azotoformans* ESR4 (6.67 plant^–1^), *P. poae* ESR6 (6.67 plant^–1^), and *S. maltophilia* ESR20 (6.33 plant^–1^). In total, inoculation with all the biofilm-producing PGPR resulted in an increase in the number of primary branches, though in varying degrees.

Similarly, the number of leaves plant^–1^ incredibly amplified in *P. azotoformans* ESR4-inoculated plants (50% increase compared to non-inoculated plants), which was not substantially different to *B. aryabhattai* ESB6 (46.7%), *P. poae* ESR6 (36.9%), and *S. maltophilia* ESR20 (34.8%). Also, the application of other PGPR increased the number of leaves. Comparably, also the leaf width was higher in those plants treated with the bacteria as compared to the non-inoculated plants.

The root and shoot dry matter plant^–1^ were also significantly and remarkably higher after the application of the different biofilm-producing rhizobacterial strains. The highest root dry matter weight increase plant^–1^ (2.89 g) was obtained in *P. azotoformans* ESR4-inoculated plants (49.7% increase compared to non-inoculated plants). Interestingly, inoculation with the same strain also improved the shoot dry matter weight plant^–1^ by 30.1%, though the increase was even higher when the plants have been treated with *B. aryabhattai* ESB6 (49.5% increase as compared to the non-inoculated plants). In this study, the largest bacterial populations were detected, as expected, in the rhizosphere (roots along with soil) of bacterized plants and not in the non-inoculated plants.

### Expression of Biochemical Traits

#### Relative Water Content (RWC)

The leaf RWC ominously fluctuated with or without the bacterial inoculation (Table 6). *P. chlororaphis* ESR15-inoculated tomato leaves exhibited the highest RWC (85.7%), whereas the *P. poae* ESR6-inoculated tomato leaves displayed the lowest RWC (57.01%).

**TABLE 6:** Relative water content, leaf pigments, oxidative stress markers, proline, and catalase in leaves of tomato plants under water deficit-stressed conditions.

		Leaf pigments (mg g^–^^1^ FW)				Oxidative stress markers			
				
Treatments	***Relative water content (%)	***Chlorophyll *a*	^α^Chlorophyll *b*	***Total chlorophyll	***Carotenoids	***Malondialdehyde (μmol g^–^^1^ FW)	**Electrolyte leakage (%)	***Proline content (μg g^–^^1^ FW)	***Catalase (U mg^–^^1^ protein)
T_1_	59.542 ± 6.87 e	1.635 ± 0.01 c	0.469 ± 0.02 c	2.104 ± 7.49E–03 e	5.300 ± 0.63 f	116.77 ± 2.54 ab	24.91 ± 1.67 a	30.47 ± 1.52 b	134.70 ± 4.24 e
T_2_	63.278 ± 4.10 de	2.181 ± 0.03 a	1.084 ± 0.04 a	3.264 ± 7.33E–02 a	8.050 ± 0.16 a	130.72 ± 0.25 a	24.14 ± 3.94 a	25.92 ± 2.07 c	161.20 ± 3.53 c
T_3_	57.075 ± 8.32 e	1.832 ± 0.19 b	0.659 ± 0.36 bc	2.491 ± 1.73E–01 d	6.135 ± 0.01 e	136.92 ± 2.3 a	22.09 ± 1.68 ab	38.25 ± 1.49 a	166.99 ± 2.12 c
T_4_	70.812 ± 4.51 cd	1.836 ± 0.09 b	0.758 ± 0.11 abc	2.595 ± 2.22E–02 d	7.007 ± 0.10 d	60.45 ± 2.2 de	13.88 ± 0.38 cd	11.18 ± 0.63 ef	148.94 ± 4.24 d
T_5_	82.947 ± 5.72 a	2.143 ± 0.03 a	1.039 ± 0.06 ab	3.181 ± 3.19E–02 ab	7.822 ± 0.01 ab	81.12 ± 0.77 cd	15.40 ± 0.88 cd	13.04 ± 0.80 de	160.00 ± 5.65 c
T_6_	85.742 ± 3.91 a	2.140 ± 0.04 a	0.843 ± 0.29 abc	2.982 ± 2.47E–01 bc	7.564 ± 0.05 bc	49.60 ± 1.4 e	12.13 ± 1.71 d	11.36 ± 1.42 ef	161.01 ± 4.24 c
T_7_	74.071 ± 4.55 bc	2.095 ± 0.06 a	1.110 ± 0.08 a	3.205 ± 1.52E–02 ab	7.502 ± 0.01 c	98.68 ± 1.7 bc	18.42 ± 3.48 bc	23.31 ± 1.47 c	188.78 ± 7.77 b
T_8_	72.714 ± 6.86 c	2.068 ± 0.02 a	0.821 ± 0.09 abc	2.889 ± 6.84E–02 c	7.579 ± 0.22 bc	60.45 ± 2.1 de	16.37 ± 1.49 cd	15.94 ± 1.00 d	164.94 ± 4.94 c
T_9_	81.021 ± 7.28 ab	2.103 ± 0.02 a	0.947 ± 0.06 ab	3.050 ± 8.45E–02 abc	7.613 ± 0.21 bc	80.85 ± 1.34 cde	15.62 ± 2.24 cd	9.31 ± 1.72 f	231.81 ± 3.53 a

T_1_ = Without bacterial inoculation, T_2_, T_3_, T_4_, T_5_, T_6_, T_7_, T_8_, and T_9_ = Tomato plants inoculated with P. azotoformans ESR4, P. poae ESR6, P. gessardii ESR9, P. cedrina ESR12, P. chlororaphis ESR15, S. maltophilia ESR20, P. veronii ESR21, and B. aryabhattai ESB6, respectively. Data are means ± standard deviations. Different letters in column imply significant difference among treatments. ***, **, *, and ^α^ indicate significant difference at 0.1% (P ≤ 0.001), 1% (P ≤ 0.01), 5% (P ≤ 0.05), and 10% (P ≤ 0.1), respectively.

#### Leaf Pigments

Beside the plant growth parameters, also the pigment production provides an indication of plant health. Overall, the tomato plants treated with different bacterial strains produced higher amounts of chl *a*, chl *b*, and total chl compared to the non-inoculated plants (Table 6). *P. azotoformans* ESR4-inoculated plants produced more chl *a* (2.18 mg g^–1^ FW), which was not considerably different to *P. cedrina* ESR12 (2.14 mg g^–1^ FW), *P. chlororaphis* ESR15 (2.14 mg g^–1^ FW), *S. maltophilia* ESR20 (2.09 mg g^–1^ FW), *P. veronii* ESR21 (2.06 mg g^–1^ FW), and *B. aryabhattai* ESB6 (2.10 mg g^–1^ FW). *S. maltophilia* ESR20-inoculated plants exhibited the highest amount of chl *b* (1.11 mg g^–1^ FW) followed by *P. azotoformans* ESR4 (1.08 mg g^–1^ FW), *P. cedrina* ESR12 (1.03 mg g^–1^ FW), *B. aryabhattai* ESB6 (0.95 mg g^–1^ FW), *P. chlororaphis* ESR15 (0.84 mg g^–1^ FW), *P. veronii* ESR21 (0.82 mg g^–1^ FW), *P. gessardii* ESR9 (0.76 mg g^–1^ FW), and *P. poae* ESR6 (0.66 mg g^–1^ FW). Interestingly, total chl was incredibly increased by 55.1, 18.4, 23.3, 51.1, 41.7, 52.3, 37.3, and 44.9% by application with *P. azotoformans* ESR4, *P. poae* ESR6, *P. gessardii* ESR9, *P. cedrina* ESR12, *P. chlororaphis* ESR15, *S. maltophilia* ESR20, *P. veronii* ESR21, and *B. aryabhattai* ESB6, respectively, as compared to that of non-inoculated plants. The carotenoid content increased after inoculation of *P. azotoformans* ESR4, *P. poae* ESR6, *P. gessardii* ESR9, *P. cedrina* ESR12, *P. chlororaphis* ESR15, *S. maltophilia* ESR20, *P. veronii* ESR21, and *B. aryabhattai* ESB6 by 51.8, 15.7, 32.2, 47.5, 42.7, 41.5, 43.0, and 43.6% increase, respectively, compared to non-inoculated plants.

#### Production of Malondialdehyde (MDA) and Electrolyte Leakage (EL)

The level of MDA represents the lipid peroxidation of membrane lipids and designates as a marker of oxidative injury. In this study, non-inoculated plant leaves accumulated MDA around 116.77 ± 2.54 μmol g^–1^ FW. Importantly, MDA accumulation was lowered by 15.5% when inoculated with S. *maltophilia* to up to 57.5% for *P. chlororaphis* ESR15 (Table 6). However, a significantly higher amount of MDA was found in plants inoculated with *P. poae* ESR6 (136.9 ± 2.3 μmol g^–1^ FW), followed by *P. azotoformans* ESR4 (130.7 ± 0.25 μmol g^–1^ FW) than non-inoculated plants (116.77 ± 2.54 μmol g^–1^ FW) (Table 6).

The electrolyte leakage is a measure of the presence of dead cells. Due to the water-deficit stress, the EL in non-inoculated plants was very high (24.9%) though not statistically distinct from plants treated with *P. azotoformans* ESR4 (24.1%) or *P. poae* ESR6 (22%). Treatment with *P. chlororaphis* ESR15 even reduced the leaf EL by 51.3% (Table 6).

### Leaf Proline and Catalase Activity

In response to water stress, proline accumulation is common in plant leaves for osmotic adjustment. Also, proline acts as a protective agent of enzymes and antioxidants. In this study, application of *P. poae* ESR6 resulted in a significant increase in proline content (38.2 μg g^–1^ FW) followed by non-inoculated plants (30.4 μg g^–1^ FW) (Table 6). However, proline content was reduced by 69.4, 63.3, 62.7, 57.2, 47.7, and 23.5% by inoculation of *B. aryabhattai* ESB6, *P. gessardii* ESR9, *P. chlororaphis* ESR15, *P. cedrina* ESR12, *P. veronii* ESR21, and *S. maltophilia* ESR20, respectively, as compared to non-inoculated plants. The catalase (CAT) activity, another plant protectant enzyme, remarkably changed by application of rhizobacteria (Table 6). The CAT activity was increased by 10.6% for *P. gessardii* ESR9 to up to 72.1% when inoculated with *B. aryabhattai*, respectively, as compared to non-inoculated plants.

## Discussion

In this study, 21 (26.9%) from 78 analyzed rhizobacterial strains isolated from rhizosphere of tomato plants were found to form biofilms (Figure 1A). Among them, 13 strains (61.9%) were identified as *Pseudomonas* comprising 8 species [*P. fluorescens* (ESR7 and ESR25), *P. azotoformans* (ESR4), *P. chlororaphis* (ESR3 and ESR15), *P. poae* (ESR6), *P. gessardii* (ESR9), *P. cedrina* (ESR12, ESR16, and ESR23), *P. veronii* (ESR13 and ESR21), and *P. parafulva* ESB18] and 6 strains (28.6%) were *Bacillus* with 4 species [*B. cereus* (ESD3, ESD21, and ESB22), *B. horikoshii* (ESD16), *B. aryabhattai* (ESB6), and *B. megaterium* (ESB9)] (Table 1). *Pseudomonas* and *Bacillus* are well-known plant-growth promoters (Kumar et al., 2012; Backer et al., 2018; Chandra et al., 2018; Naseem et al., 2018; Jochum et al., 2019). Among the identified rhizobacteria, *B. cereus* (Yan et al., 2017), *P. chlororaphis* (Selin et al., 2010), and *P. fluorescens* (Ude et al., 2006) were only reported to form biofilms under laboratory conditions. Thus, several novel biofilm-producing rhizobacterial strains were identified in this current study. All these biofilm-producing rhizobacterial strains are nonpathogenic to human and animals based on the hemolytic test using 5% sheep blood (data not shown).

The characterized rhizobacterial biofilm matrices (i.e., EPS) predominantly contain proteins, polysaccharides, nucleic acids, and lipids (Figure 3). The protein peaks (amide I, II, and III) were much higher than the peaks of polysaccharides, nucleic acids, and lipids (Figure 3). Our results align with the results of Conrad et al. (2003). Indeed, these protein components show the characteristic IR band through C = O stretching at the amide I region, in contrast to the C-N bending and the N–H stretching at the amide II region and the amide III region (Naumann, 2001; Ojeda et al., 2008; Haque et al., 2014; Mosharaf et al., 2018). On the other hand, the band region of polysaccharides principally resulted from a stretching vibration of C–C and C–O bonds and the deformation of C–O–H and C–O–C bonds (Naumann, 2001). Negatively charged functional groups of biofilm matrices were reported to bind with heavy metals, leading to heavy metal remediation from the contaminated environment (Teitzel and Parsek, 2003; Xie et al., 2015). Moreover, various functional groups play a pivotal role in bacterial root colonization (Gupta et al., 2017).

In the current study, a significant number of rhizobacteria synthesized IAA (90.5%), fixed N_2_ (100%), solubilized nutrients [P (85.7%), K (42.8%), and Zn (61.9%)], produced siderophores (76.2%), volatile compounds [e.g., acetoin (9.5%), indole (47.6%), ammonia (95.2%), and hydrogen cyanide (14.3%)], and hydrolytic enzymes [e.g., ACC deaminase (100%), catalases (100%), oxidases (76.19%), gelatinases (80.95%), arginine dehydrolases (76.2%), lipases (85.7%), cellulases (100%), and proteases (47.6%)] (Tables 2–4 and Figure 7). From the identified and here characterized bacteria, *P. cedrina* ESR12, *P. chlororaphis* ESR15, and *B. cereus* ESD3 inhibited the growth of *X. campestris* pv. *campestris* ATCC 33913 (Figure 8), while growth of *R. solanacearum* ATCC^®^ 11696^TM^ was only controlled by *P. chlororaphis* ESR15 and *B. cereus* ESD21 (Figure 8). Accordingly, several biocontrol-related traits, such as production of siderophores, ammonia, HCN, and several hydrolytic enzymes, were also present in *P. cedrina* ESR12, *P. chlororaphis* ESR15, and *B. cereus* (ESD3 and ESD21) (Table 3). Therefore, these identified bacterial strains can be used even under field conditions to control these tomato pathogens, thus preventing yield lost. Taken together, our results suggest that all these biofilm-producing rhizobacterial strains are PGPR expressing multiple PGP traits. Beneficial bacterial biofilms were reported to attach to the plant roots (e.g., rice, wheat, maize, cucumber, and various legumes) and participate in cycling of nutrients and control of phytopathogens, leading to an increase in the productivity of crops (Nieuwenhove et al., 2000; Yang et al., 2004; Kim et al., 2005; Williams et al., 2008; Fan et al., 2011).

The here analyzed extracellular polymeric substances (EPS) are highly hydrated polymers synthesized by different microorganisms including bacteria (Costa et al., 2018). The contents of the EPS play many important roles such as adhesion, soil aggregation, cohesion, retention of water, protective barrier, absorption of organic compounds, absorption of inorganic ions, nutrient source, exchange of genetic information, electron donor or acceptor, export of cell components, sink of excess energy, and the binding of enzymes (Flemming and Wingender, 2010). Hepper (1975) reported that bacterial EPS creates a microenvironment that holds water and dries up more slowly, thus protecting the bacteria and plant roots against extreme desiccation. Furthermore, Roberson and Firestone (1992) have shown that EPS from *Pseudomonas* strains increased the water-holding capacity in sandy soil. EPS released from biofilms also improved the permeability by creating soil aggregation and maintaining a higher water potential around the roots, thereby increasing the uptake of nutrients by plants and protecting them from water shortage (Selvakumar et al., 2012). For example, inoculation of sunflower seedlings with EPS-producing *P. putida* GAP-P45 showed an augmented plant growth and increased the survival rates under drought-stressed condition (Sandhya et al., 2009). EPS production was reported to be positively correlated with drought tolerance of cowpea (Rodríguez-Navarro et al., 2007) and foxtail millet (Niu et al., 2018). In our present study, we observed that rhizobacterial biofilm matrices, i.e., EPS, are sponge-like and fibrous (Figure 5). Thus, these rhizobacterial EPS may hold water and/or increase the water-holding capacity in the soil to assist the bacteria and the plant roots under water-deficit stress. In the present study, inoculation of tomato plants with biofilm-producing PGPR increased plant growth (Table 5) and resulted in more water-deficit stress tolerance than non-inoculated plants (Figure 9).

Plant growth-promoting rhizobacteria produce the phytohormones which are directly involved in plant growth promotion (Patten and Glick, 1996). Among these phytohormones, IAA increases the number of root hairs and lateral roots, and this increased root surface area promotes nutrient and water uptake by the plants even under drought stress (Gray and Smith, 2005; Mantelin and Touraine, 2009; Ruzzi and Aroca, 2015; Saikia et al., 2018; Ojuederie et al., 2019). Thus, a constitutive synthesis of IAA is required for proper plant growth and development. In contrast, primary root length is stimulated at low concentrations of IAA, while increased at high IAA concentration promotes lateral root formation but decreased primary root lengths (Vacheron et al., 2013). Ethylene, another phytohormone, also controls different physiological responses including senescence, ripening of fruits, initiation of roots, and the abscission and inhibition of storage organ formation. Overproduction of ethylene significantly reduces the root and shoot development (Glick et al., 1998). However, ACC deaminases in PGPR reduced the ethylene production leading to an increased crop productivity under water deficit-stressed conditions (Saikia et al., 2018; Ojuederie et al., 2019). In this study, biofilm-producing PGPR expressed IAA and ACC deaminases *in vitro* (Tables 2, 3). Importantly, *in vivo* grown tomato plants bacterized with biofilm-producing *P. azotoformans* ESR4, *P. poae* ESR6, *P. gessardii* ESR9, *P. cedrina* ESR12, *P. chlororaphis* ESR15, *S. maltophilia* ESR20, *P. veronii* ESR21, and *B. aryabhattai* ESB6 showed an enhanced plant growth in terms of plant height, number of primary branches, number of leaves, maximum leaf length and width, root and shoot dry matter production (Table 6), and withstanding water-deficit stress (Figure 9). Therefore, the isolated PGPR are capable of attenuating water-deficit stress by synthesizing IAA and ACC deaminases, thereby reducing the accumulation of ethylene in tomato.

Leaf RWC is the index of physiological water status of plants that improves their tolerance to drought stress (Kadioglu et al., 2011). Reduction of leaf RWC was reported to positively correlate with closure of stomata which leads to decreased CO_2_ assimilation in plants (Wezel et al., 2014). Under severe drought stress, a reduced RWC decreased leaf pigments such as chl *a* and *b*, resulting in a reduced photosynthesis rate (Kawamitsu et al., 2000; Manivannan et al., 2007; Mafakheri et al., 2010). However, in stressed plants, increased chlorophyll concentration can be used as an index of tissue tolerance (Lutts et al., 1996). Heidari and Golpayegani (2012) reported that leaf chlorophylls were increased in basil (*Ocimum basilicum*) after inoculation of PGPR even under water deficit-stressed conditions. Saline or water stress-induced osmotic conditions enhanced the chloroplast development (Chang et al., 1997). In this study, a significant reduction of leaf RWC was observed in non-inoculated plants, while it only slightly decreased in plants inoculated with *P. azotoformans* ESR4 and *P. poae* ESR6. However, all other PGPR-inoculated plants did not show any reduction in leaf RWC (Table 6). It was also observed that the photosynthetic leaf pigment contents (such as chl *a*, chl *b*, and total chl) in all PGPR-inoculated plants were remarkably higher than in non-inoculated plants (Table 6). Therefore, biofilm-producing PGPR efficiently retained the leaf RWC and the photosynthetic efficiency of plants under water-limiting conditions.

In the presence of environmental stimuli, i.e., drought, high temperature, and salt, plant cells suffer oxidative stress by lipid peroxidation resulting in tissue damages (Catal, 2006). MDA is a product of lipid peroxidation and is used as an indicator of oxidative stress (Esterbauer and Cheeseman, 1990). In this study, non-inoculated plants and the plants inoculated with *P. azotoformans* ESR4 and *P. poae* ESR6 produced more MDA as compared to other biofilm-producing PGPR-treated plants (Table 6). Similar trends were also observed for electrolyte leakage, a measure for cell death. Proline is one of the common compatible osmoprotectants and a stress marker which influences adaptive responses (Maggio et al., 2002; Mafakheri et al., 2010). This component shields cells from dehydration, detoxifies stressed cells from ROS, maintains the integrity of subcellular structures and enzymes, and protects the transcriptional and translational machinery of plants (Maggio et al., 2002). In this study, *P. poae* ESR6-inoculated plants synthesized remarkably higher amounts of proline which was statistically similar with non-inoculated plants, suggesting that non-inoculated- and *P. poae* ESR6-inoculated plants are more stressed than other plants (Table 6). The enzymatic and non-enzymatic defense system showed that PGPR-inoculated plants accumulated under water-deficit stress more CAT and carotenoids than non-inoculated control plants (Table 6). Kohler et al. (2008) reported that inoculation with *P. mendocina* and *Glomus intraradices* significantly increased CAT in lettuce plants under severe drought conditions. Therefore, the experimental results showed that our identified biofilm-producing PGPR attenuate water-deficit stress and promoted plant growth which might be associated with multiple mechanisms such as biofilm formation, production of EPS, synthesis of IAA and ACC deaminases, increased enzymatic and non-enzymatic defense systems, and the improved solubilization of nutrients.

## Conclusion

The tomato plants grown in drought-prone ecosystems of Bangladesh are associated with a variety of rhizobacteria with multifarious plant growth-promoting traits. Thus, all these biofilm PGPR can be used as biofertilizers, plant-growth promoters, suppressors of phytopathogens, and alleviators of abiotic stressors, such as drought, salinity, and heat. Application of biofilm PGPR would reduce environmental pollution as well as global warming leading to climate change. Thus, it would be necessary in the near future to further apply phenotypic screening of bacterial strains and their ability to form biofilms as a rapid selection criterion of PGPR. Our future studies should focus on biofilm formation in PGPR under field conditions.

## Data Availability Statement

The datasets presented in this study can be found in online repositories. The names of the repository/repositories and accession number(s) can be found in the article/Supplementary Material.

## Author Contributions

MMH conceived the idea, developed the methodologies, conducted several experiments, wrote the manuscript and collected the research fund. MKM conducted siderophore, K solubilization assays, antagonistic tests, pot experiments and analyzed the data. MK collected the samples and characterized the strains. MAH helped in planning the research and conducted the FTIR spectroscopy. MSB determined MDA, protein and catalase activity. MSI isolated bacterial DNA, identified the bacteria based on 16S rRNA gene sequencing and constructed phylogenetic tree. MMI took the SEM images. HBS and MAS verified and quantified the IAA using HPLC. MMUM and AHM supervised MS student (MK). All the authors read the manuscript.

## Conflict of Interest

The authors declare that the research was conducted in the absence of any commercial or financial relationships that could be construed as a potential conflict of interest.

## Footnotes

1 http://www.ncbi.nlm.nih.gov/

2 http://www.ncbi.nlm.nih.gov/Blast.cgi

## Supplementary Material

Supplementary Figure 1 Production of auxins by different rhizobacterial strains. Initially each rhizobacterium was grown in LB broth supplemented with 0.2% of L-tryptophan in agitating condition. After 48 h incubation, 1 mL culture was collected and centrifuged. Then 500 μL supernatant was mixed with 1 mL of Salkowski reagent. Development of pink color indicated positive for auxin related compounds including IAA production.

Supplementary Figure 2 Expression of *nifH* gene in different rhizobacterial strains. Primer pairs of Ueda19F and Ueda19R amplified 389 bp, while 310 pb was amplified by KAD3-F and DVV-R primer pairs. **(A)** Lane 1- ESR3, Lane 2- ESR4, Lane 3- ESR6, Lane 4- ESR7, Lane 5- ESR9, Lane 6- 1 kb+ ladder, Lane-7 ESD3, Lane-8 ESD8, Lane-9 ESD16 23, Lane-10 ESD21, Lane-10 ESB9, Lane-12 Negative control; Lane 1 to 5 used KAD3-F and DVV-R primers pair and lane 7-11 used Ueda19F and Ueda407R **(B)** Lane 1- ESR12, Lane 2- ESR13, Lane 3- ESR15, Lane 4- ESR16, Lane 5- ESR20, Lane 6- 1 kb+ ladder, Lane-7 ESR21, Lane-8 ESR23, Lane-9 ESB18, Lane-10 ESB22 72, Lane-11 ESR20, Lane-12 Negative control; Lane 1 to 5 used Ueda19F and Ueda407R primers pair and lane 7-11 used KAD3F and DVVR primers pair.

Supplementary Figure 3 Qualitative ammonia production by different rhizobacterial strains. The 50 μL (10^8^ CFU mL^–1^) culture were inoculated in the glass test tubes containing 5 mL peptone water and incubated at 28°C for 72 h. Then 1 mL Nessler’s reagent was added. Development of yellow to brown color indicated positive results.

Supplementary Figure 4 Qualitative production of HCN. Rhizobacterial strains were streaked on LB agar plates containing 0.45% glycine. Then filter papers dipped in alkaline pirate solution and placed on the lids of petri plates, sealed with parafilm and incubated at 28°C for 24 h. Positive HCN production resulted in a color development from yellow to reddish-brown.

Supplementary Figure 5 Production of biofilms by some selected rhizobacterial strains after 72 h incubation at 28°C in stationary condition. Biofilm formation on SOBG without 25% PEG 6000 **(A)** and SOBG with 25% PEG 6000 **(B)**.

## References

[B1] Ahemad M. Kibret M. (2014). Mechanisms and applications of plant growth promoting rhizobacteria: current perspective. *J. King Saud Univ. Sci.* 26 1–20. 10.1016/j.jksus.2013.05.001

[B2] Alamgir M. Mohesnipour M. Homsi R. Wang X. Shahid S. Shiru M. S. (2019). Parametric assessment of seasonal drought risk to crop production in Bangladesh. *Sustainability* 11:1442 10.3390/su11051442

[B3] Ando S. Goto M. Meunchang S. Thongra-ar P. Fujiwara T. Hayashi H. (2005). Detection of *nifH* sequences in surgacane (*Saccharum officinarum* L.) *and pineapple (Ananas comosus L.) Soil Sci*. *Plant Nutr.* 51 303–308. 10.1111/j.1747-0765.2005.tb00034.x

[B4] Backer R. Rokem J. S. Ilangumaran G. Lamont J. Praslickova D. Ricci E. (2018). Plant growth-promoting rhizobacteria: context, mechanisms of action, and roadmap to commercialization of biostimulants for sustainable agriculture. *Front. Plant Sci.* 9:1473. 10.3389/fpls.2018.01473 30405652 PMC6206271

[B5] Bais H. P. Fall R. Vivanco J. M. (2004). Biocontrol of *Bacillus subtilis* against infection of Arabidopsis roots by *Pseudomonas syringae* is facilitated by biofilm formation and surfactin production. *Plant Physiol.* 134 307–319. 10.1104/pp.103.028712 14684838 PMC316310

[B6] Barker D. E. Surh N. H. (1982). “Atomic absorption and flame emission spectroscopy,” in *Methods of soil analysis, part 2: Chemical and Microbiological Properties*, eds Page A. L. Miller R. H. Keeney D. R. (Madison, WI: American Society of Agronomy), 13–26.

[B7] Bates L. S. Waldren R. P. Teare I. D. (1973). Rapid determination of free proline for water-stress studies. *Plant Soil* 39 205–207. 10.1007/BF00018060

[B8] Bremner J. M. Mulvaney C. S. (1982). “Total nitrogen,” in *Methods of Soil Analysis, Part 2: Chemical and Microbiological Properties*, eds Page A. L. Miller R. H. Keeney D. R. (Madison, WI: American Society of Agronomy), 595–624.

[B9] Catal A. (2006). An overview of lipid peroxidation with emphasis in outer segments of photoreceptors and the chemiluminescence assay. *Int. J. Biochem. Cell Biol.* 38 1482–1495. 10.1016/j.biocel.2006.02.010 16621670

[B10] Chandra D. Srivastava R. Click B. R. Sharma A. K. (2018). Drought-tolerant *Pseudomonas* spp. Improve the growth performance of finger millet *(Eleusine coracana L.)* Gaertn under non-stressed and drought-stressed conditions. *Pedosphere* 28 227–240. 10.1016/S1002-0160(18)60013-X

[B11] Chang C.-C. Locy R. D. Smeda R. Sahi S. V. Singh N. K. (1997). Photoautotrophic tobacco cells adapted to grow at high salinity. *Plant Cell Rep.* 16 495–502. 10.1007/BF01092773 30727639

[B12] Conrad A. Kontro M. Keinänen M. M. Cadoret A. Faure P. Mansuy-Huault L. (2003). Fatty acids of lipid fractions in extracellular polymeric substances of activated sludge flocs. *Lipids* 38 1093–1105. 10.1007/s11745-006-1165-y 14669975

[B13] Costa O. Y. A. Raaijmakers J. M. Kuramae E. E. (2018). Microbial extracellular polymeric substances: ecological function and impacts on soil aggregation. *Front. Microbial.* 9:1636. 10.3389/fmicb.2018.01636 30083145 PMC6064872

[B14] Dinesh R. Srinivasan V. Hamza S. Sarathambal C. Anke Gowda S. J. Ganeshamurthy A. N. (2018). Isolation and characterization of potential Zn solubilizing bacteria from soil and its effects on soil Zn release rates, soil available Zn and plant Zn content. *Geoderma* 321 173–186. 10.1016/j.geoderma.2018.02.013

[B15] Du J. Chen X. Li W. Gao Q. (2004). Osmoregulation mechanism of drought stress and genetic engineering strategies for improving drought resistance in plants. *For. Stud. China* 6 56–62. 10.1007/s11632-004-0021-5

[B16] Dworkin M. Foster J. (1958). Experiments with some microorganisms which utilize ethane and hydrogen. *J. Bacteriol.* 75 592–603. 10.1128/jb.75.5.592-603.1958 13538930 PMC290115

[B17] Dye D. W. (1968). A taxonomic study of the genus *Erwinia.* I. The Amylovora group. *New Zealand J. Sci.* 11 590–607.

[B18] Esterbauer H. Cheeseman K. H. (1990). Determination of aldehydin lipid peroxidation products: malondehyde and 4-hydroxynonenal. *Methods Enzymol.* 186 407–421. 10.1016/0076-6879(90)86134-h 2233308

[B19] Fan B. Chen X. H. Budiharjo A. Bleiss W. Vater J. Borriss R. (2011). Efficient colonization of plant roots by the plant growth promoting bacterium *Bacillus amyloliquefaciens* FZB42, engineered to express green florescent protein. *J. Biotechnol.* 151 303–311. 10.1016/j.jbiotec.2010.12.022 21237217

[B20] Fertilizer Recommendation Guide [Frg]. (2012). *Fertilizer Recommendation Guide. Bangladesh Agricultural Research Council (BARC).* Dhaka: Farmgate.

[B21] Flemming H. C. Wingender J. (2010). The biofilm matrix. *Nat. Rev. Microbiol.* 8:623. 10.1038/nrmicro2415 20676145

[B22] Furuya N. Yamasaki S. Nishioka M. Shirasshi I. Ityama K. Matsuyama N. (1997). Antibacterial activities of Pseudomonads against plant pathogenic organisms and efficacy of *Pseudomonas aeruginosa* ATCC7700 against bacterial wilt of tomato. *Ann. Phytopathol. Soc. Jpn.* 63 417–424. 10.3186/jjphytopath.63.417

[B23] Glick B. R. Penrose D. M. Li J. A. (1998). A model for lowering plant ethylene concentrations by plant growth promoting rhizobacteria. *J. Theorit. Biol.* 190 63–68. 10.1006/jtbi.1997.0532 9473391

[B24] Glickmann E. Dessaux Y. (1995). A critical examination of the speci^®^ city of the Salkowski reagent for indolic compounds produced by phytopathogenic bacteria. *Appl. Environ. Microbiol.* 6 793–796. 10.1128/aem.61.2.793-796.1995 16534942 PMC1388360

[B25] Gordon S. A. Weber R. P. (1951). Colorimetric estimation of indole acetic acid. *Plant Physiol.* 26 192–195. 16654351 10.1104/pp.26.1.192 PMC437633

[B26] Gouda S. Kerry R. G. Das G. Paramithiotis S. Shin H.-S. Patra J. K. (2018). Revitalization of plant growth promoting rhizobacteria for sustainable development in agriculture. *Microbiol. Res.* 206 131–140. 10.1016/j.micres.2017.08.016 29146250

[B27] Gray E. J. Smith D. L. (2005). Intracellular and extracellular PGPR: commonalities and distinctions in the plant–bacterium signaling processes. *Soil Biol. Biochem.* 37 395–412. 10.1016/j.soilbio.2004.08.030

[B28] Gupta S. Kaushal R. Spehia R. S. Pathania S. S. Sharma V. (2017). Productivity of capsicum influenced by conjoint application of isolated indigenous PGPR and chemical fertilizers. *J. Plant Nutr.* 40 921–927. 10.1080/01904167.2015.1093139

[B29] Habiba U. Shaw R. (2013). Drought scenario in Bangladesh. In water insecurity: a social dilemma (Community, Environment and Disaster Risk Management). *Emerald Group Publ. Ltd.* 13 213–245. 10.1108/S2040-726220130000013016

[B30] Haque M. A. Aldred P. Chen J. Barrow C. Adhikari B. (2014). Drying and denaturation characteristics of α-lactalbumin, β-lactoglobulin, and bovine serum albumin in a convective drying process. *J. Agril. Food Chem.* 62 4695–4706. 10.1021/jf405603c 24819828

[B31] Haque M. M. Hirata H. Tsuyumu S. (2012). Role of PhoP–PhoQ two-component system in pellicle formation, virulence and survival in harsh environments of *Dickeya dadantii* 3937. *J. Gen. Plant Pathol.* 78 176–189. 10.1007/s10327-012-0372-z

[B32] Haque M. M. Hirata H. Tsuyumu S. (2015). SlyA regulates *motA* and *motB*, virulence and stress-related genes under conditions by the PhoP–PhoQ two-component system in *Dickeya dadantii* 3937. *Res. Microbiol.* 166 467–475. 10.1016/j.resmic.2015.05.004 26027774

[B33] Haque M. M. Kabir M. S. Aini L. Q. Hirata H. Tsuyumu S. (2009). SlyA, a MarR family transcriptional regulator, is essential for virulence in *Dickeya dadantii* 3937. *J. Bacteriol.* 191 5409–5419. 10.1128/JB.00240-09 19542281 PMC2725626

[B34] Haque M. M. Oliver M. M. H. Nahar K. Alam M. Z. Hirata H. Tsuyumu S. (2017). CytR homolog of *Pectobacterium carotovorum* subsp. carotovorum controls air-liquid biofilm formation by regulating multiple genes involved in cellulose production, c-di-GMP signaling, motility, and type III secretion system in response to nutritional and environmental signals. *Front. Microbiol.* 8:972. 10.3389/fmicb.2017.00972 28620360 PMC5449439

[B35] Hayward A. C. (1992). *Identification of Pseudomonas solanacearum*. In: SAVERNET Bacterial wilt training course. Taiwa: AVRDC, 101.

[B36] Heidari M. Golpayegani A. (2012). Effects of water stress and inoculation with plant growth promoting rhizobacteria (PGPR) on antioxidant status and photosynthetic pigments in basil (*Ocimum basilicum* L.). *J. Saudi Soc. Agril. Sci.* 11 57–61. 10.1016/j.jssas.2011.09.001

[B37] Hepper C. M. (1975). “Extracellular polysaccharides of soil bacteria,” in *Soil microbiology. A critical review*, ed. Walker N. (New York, NY: Wiley), 99–111.

[B38] Hu X.-B. Xu K. Wang Z. Ding L.-L. Ren H.-Q. (2013). Characteristics of biofilm attaching to carriers in moving bed biofilm reactor used to treat vitamin C wastewater. *Scanning* 35 283–291. 10.1002/sca.21064 23168685

[B39] Intergovernmental Panel on Climate Change [Ipcc]. (2014). “Climate change 2014: Synthesis report,” in *Contribution of working groups I, II and III to the fifth assessment report of the Intergovernmental Panel on Climate Change*, eds Core Writing Team, Pachauri R. K. Meyer L. A. (Geneva: IPCC), 151.

[B40] Jahn C. E. Selimi D. A. Barak J. D. Charkowski A. O. (2011). The *Dickeya dadantii* biofilm matrix consists of cellulose nanofibres, and is an emergent property dependent upon the type III secretion system and the cellulose synthesis operon. *Microbiology* 157 2733–2744. 10.1099/mic.0.051003-0 21719543

[B41] Jarecki M. K. Parkin T. B. Chan A. S. Hatfield J. L. Jones R. (2008). Greenhouse gas emissions from two soils receiving nitrogen fertilizer and swine manure slurry. *J. Environ. Qual.* 37 1432–1438. 10.2134/jeq2007.0427 18574174

[B42] Jochum M. D. McWilliams K. L. Borrego E. J. Kolomiets M. V. Niu G. Pierson E. A. (2019). Bioprospecting plant growth-promoting rhizobacteria that mitigate drought stress in grasses. *Front. Microbiol.* 10:2016. 10.3389/fmicb.2019.02106 31552009 PMC6747002

[B43] Jones Jr. J. B Case V. W. (1990). “Sampling, handling, and analyzing plant tissue samples,” in *Soil testing and plant analysis*, 3rd Edn, ed. Westermaan W. S. (Madison, WI: American Society of Agronomy), 389–427. 10.2136/sssabookser3.3ed.c15

[B44] Kadioglu A. Saruhan N. Saǧlam A. Terzi R. Acet T. (2011). Exogenous salicylic acid alleviates effects of long term drought stress and delays leaf rolling by inducing antioxidant system. *Plant Growth Regul.* 64 27–37. 10.1007/s10725-010-9532-3

[B45] Karmakar R. Das I. Dutta D. Rakshit A. (2016). Potential effects of climate change on soil properties: a review. *Sci. Intl.* 4 51–73. 10.3923/sciintl.2016.51.73

[B46] Kaushal M. Wani S. P. (2016). Plant-growth-promoting rhizobacteria: drought stress alleviators to ameliorate crop production in drylands. *Ann. Microbiol.* 66 35–42. 10.1007/s13213-015-1112-3

[B47] Kawamitsu Y. Driscoll T. Boyer J. S. (2000). Photosynthesis during desiccation in an intertidal alga and a land plant. *Plant Cell Physiol*. 41 344–353. 10.1093/pcp/41.3.344 10805598

[B48] Ker K. (2011). *A Greener Grass: Improving Biofuel Feedstock Production of Switchgrass (Panicum virgatum* L.) by Inoculation with endophytic rhizobacteria. St W, Montreal, QC: McGill University.

[B49] Khan M. Y. Haque M. M. Molla A. H. Rahman M. M. Alam M. Z. (2017). Antioxidant compounds and minerals in tomatoes by *Trichoderma*-enriched biofertilizer and their relationship with the soil environments. *J. Integr. Agric.* 15 60356–60357. 10.1016/S2095-3119(16)61350-3

[B50] Kim C. Kecskes M. L. Deaker R. J. Gilchrist K. New P. B. Kennedy I. R. (2005). Wheat root colonization and nitrogenase activity by *Azospirillum* isolates from crop plants in Korea. *Can. J. Microbiol.* 51 948–956. 10.1139/w05-052 16333334

[B51] Kohler J. Hernandez J. A. Caravaca F. Roldán A. (2008). Plant-growth promoting rhizobacteria and arbuscular mycorrhizal fungi modify alleviation biochemical mmechanisms in water-stressed plants. *Fun. Plant Biol.* 35 141–151. 10.1071/EPO7218 32688765

[B52] Kumar P. Dubey R. C. Maheshwari D. K. (2012). *Bacillus* strains isolated from rhizosphere showed plant growth promoting and antagonistic activity against phytopathogens. *Microbiol. Res.* 167 493–499. 10.1016/j.micres.2012.05.002 22677517

[B53] Lelliott R. A. Dickey R. S. (1984). “Genus VII. Erwinia,” in *Bergey’s Manual of Systematic Bacteriology*, eds Krieg N. R. Holt J. G. (Baltimore: Williams and Wilkins), 469–476.

[B54] Lorck H. (1948). Production of hydrocyanic acid by bacteria. *Physiol. Plant.* 1 142–146. 10.1111/j.1399-3054.1948.tb07118.x

[B55] Lugtenberg B. Kamilova F. (2009). Plant-growth-promoting rhizobacteria. *Annl. Rev. Microbiol.* 63 541–556. 10.1146/annurev.micro.62.081307.162918 19575558

[B56] Lutts S. Kinet J. M. Bouharmont J. (1996). NaCl-induced senescence in leaves of rice (*Oryza sativa* L.) *cultivars differing in salinity resistance*. *Ann. Bot.* 78 389–398. 10.1006/anbo.1996.0134

[B57] Mafakheri A. Siosemardeh A. Bahramnejad B. Struik P. C. Sohrabi Y. (2010). Effect of drought stress on yield, proline and chlorophyll contents in three chickpea cultivars. *Austr. J. Crop Sci.* 4 580–585.

[B58] Maggio A. Miyazaki S. Veronese P. Fujita T. Ibeas J. L. Damsz B. (2002). Does proline accumulation play an active role in stress-induced growth induction? *Plant J.* 31 699–712. 10.1046/j.1365-313X.2002.01389.x 12220262

[B59] Mah T. F. Pitts B. Pellock B. Walker G. C. Stewart P. S. O’Toole G. A. (2003). A genetic basis for *Pseudomonas aeruginosa* biofilm antibiotic resistance. *Nature* 426 306–310. 10.1038/nature02122 14628055

[B60] Mahmood S. Daur I. Al-Solaimani S. G. Ahmad S. Madkour M. H. Yasir M. (2016). Plant growth promoting rhizobacteria and silicon synergistically enhance salinity tolerance of mung bean. *Front. Plant Sci.* 7:876. 10.3389/fpls.2016.00876 27379151 PMC4911404

[B61] Mahmoudi T. R. Yu J. M. Liu S. Pierson I. I. I. L. S. Person E. A. (2019). Drought-stress tolerance in wheat seedlings conferred by phenazine-producing rhizobacteria. *Front. Microbiol.* 10:1590. 10.3389/fmicb.2019.01590 31354678 PMC6636665

[B62] Manivannan P. Abdul Jaleel C. Sankar B. Kishorekumar A. Somasundaram R. Lakshmanan G. M. A. (2007). Growth, biochemical modifications and proline metabolism in *Helianthus annuus* L. as induced by drought stress. *Colloids Surf. B: Biointerfaces* 59 141–149. 10.1016/j.colsurfb.2007.05.002 17560769

[B63] Mantelin S. Touraine B. (2009). Plant growth-promoting rhizobacteria and nitrate availability: impacts on root development and nitrate uptake. *J. Expt. Bot.* 55 27–34. 10.1093/jxb/erh010 14623902

[B64] Martins S. J. Rocha G. A. de Melo H. C. Georg R. C. Ulhôa C. J. Dianese E. C. (2018). Plant-associated bacteria mitigate drought stress in soybean. *Environ. Sci. Pollut. Res.* 25 13676–13686. 10.1007/s11356-018-1610-5 29502259

[B65] McLean E. O. (1982). “Soil pH and lime requirement,” in *Methods of soil analysis, part 2: Chemical and microbiological properties*, eds Page A. L. Miller R. H. Keeney D. R. (Madison, WI: American Society of Agronomy), 199–224.

[B66] Meenakshi, Annapurna K. Govindasamy V. Choudhary D. K. (2019). Mitigation of drought stress in wheat crop by drought tolerant endophytic bacterial isolates. *Vegetos* 32 486–493. 10.1007/s42535-019-00060-1

[B67] Milanov D. S. Prunic B. Z. Velhner M. J. Pajic M. L. Cabarkapa I. S. (2015). Rdar morphotype- a resting stage of some *Enterobacteriaceae*. *Food Feed Res.* 42 43–50. 10.5937/FFR1501043M

[B68] Mohite B. (2013). Isolation and characterization of indole acetic acid (IAA) producing bacteria from rhizospheric soil and its effect on plant growth. *J. Soil Sci. Plant Nutr.* 13 638–649. 10.4067/S0718-95162013005000051 27315006

[B69] Mosharaf M. K. Tanvir M. Z. H. Haque M. M. Haque M. A. Khan M. A. A. Molla A. H. (2018). Metal-adapted bacteria isolated from wastewaters produce biofilms by expressing proteinaceous curli fimbriae and cellulose nanofibers. *Front. Microbiol.* 9:1334. 10.3389/fmicb.2018.01334 29988579 PMC6026672

[B70] Myo E. M. Ge B. Ma J. Cui H. Liu B. Shi L. (2019). Indole-3-acetic acid production by *Streptomyces fradiae* NKZ-259 and its formulation to enhance plant growth. *BMC Microbiol.* 19:155. 10.1186/s12866-019-1528-1 31286877 PMC6615096

[B71] Naseem H. Ahsan M. Shahid M. A. Khan N. (2018). Exopolysaccharides producing rhizobacteria and their role in plant growth and drought tolerance. *J. Basic Microbiol.* 58 1009–1022. 10.1002/jobm.201800309 30183106

[B72] Naumann D. (2001). FT-infrared and FT-Raman spectroscopy in biomedical research. *Appl. Spectroscopy Rev.* 36 239–298. 10.1081/ASR-100106157

[B73] Naumann D. Barnickel G. Bradaczek H. Labischinski H. Giesbrecht P. (1982). Infrared spectroscopy, a tool for probing bacterial peptidoglycan: potentialities of infrared spectroscopy for cell wall analytical studies and rejection of models based on crystalline chitin. *Eur. J. Biochem.* 125 505–515. 10.1111/j.1432-1033.1982.tb06711.x 7117249

[B74] Nautiyal C. (1999). An efficient microbiological growth medium for screening phosphate solubilizing microorganisms. *FEMS Microbiol. Lett.* 170 265–270. 10.1111/j.1574-6968.1999.tb13383 9919677

[B75] Nelson D. W. Sommers L. E. (1982). “Total carbon, organic carbon, and organic matter,” in *Methods of soil analysis, part 2: Chemical and microbiological properties*, eds Page A. L. Miller R. H. Keeney D. R. (Madison, WI: American Society of Agronomy), 539–579.

[B76] Ngumbi E. Kloepper J. (2016). Bacterial mediated drought tolerance: current and future prospects. *Appl. Soil. Ecol.* 105 109–125. 10.1016/j.apsoil.2016.04.009

[B77] Nieuwenhove C. V. Holm V. Kulasooriya S. A. Vlassak K. (2000). Establishment of *Azorhizobium caulinodans* in the rhizosphere of wetland rice (*Oryza sativa* L.). *Biol. Fertil. Soils* 31 143–149. 10.1007/s003740050637

[B78] Niu X. Song L. Xiao Y. Ge W. (2018). Drought-tolerant plant growth-promoting rhizobacteria associated with foxtail millet in a semi-arid agroecosystem and their potential in alleviating drought stress. *Front. Microbiol.* 8:2580. 10.3389/fmicb.2017.02580 29379471 PMC5771373

[B79] Ojeda J. J. Romero-González M. E. Bachmann R. T. Edyvean R. G. Banwart S. A. (2008). Characterization of the cell surface and cell wall chemistry of drinking water bacteria by combining XPS, FTIR spectroscopy, modeling, and potentiometric titrations. *Langmuir* 24 4032–4040. 10.1021/la702284b 18302422

[B80] Ojuederie O. B. Olanrewaju O. S. Babalola O. O. (2019). Plant growth promoting rhizobacterial mitigation of drought stress in crop plants: implications for sustainable agriculture. *Agronomy* 9:712 10.3390/agronomy9110712

[B81] Pandin C. Coq D. L. Canette A. Aymerich S. Briandet R. (2017). Should the biofilm mode of the life be taken into consideration for microbial biocontrol agents? *Microb. Biotechnol*. 10 719–734. 10.1111/1751-7915.12693 28205337 PMC5481536

[B82] Patten C. L. Glick B. R. (1996). Bacterial biosynthesis of indole-3-acetic acid. *Can. J. Microbiol.* 42 207–220. 10.1139/m96-032 8868227

[B83] Penrose D. M. Glick B. R. (2003). Methods for isolating and characterizing ACC deaminase-containing plant growth-promoting rhizobacteria. *Physiol. Plant.* 118 10–15. 10.1034/j.1399-3054.2003.00086.x 12702008

[B84] Pérez-Miranda S. Cabirol N. George-Téllez R. Zamudio-Rivera L. S. Fernández F. J. (2007). O-CAS, a fast and universal method for siderophore detection. *J. Microbiol. Meth.* 70 127–131. 10.1016/j.mimet.2007.03.023 17507108

[B85] Roberson E. B. Firestone M. K. (1992). Relationship between desiccation and exopolysaccharide production in a soil *Pseudomonas* sp. *Appl. Environ. Microbiol.* 58 1284–1291. 10.1128/aem.58.4.1284-1291.1992 16348695 PMC195588

[B86] Rodríguez-Navarro D. N. Dardanelli M. S. Ruíz-Saínz J. E. (2007). Attachment of bacteria to the roots of higher plants. *FEMS Microbiol. Lett.* 272 127–136. 10.1111/j.1574-6968.2007.00761.x 17521360

[B87] Römling U. (2005). Characterization of the rdar morphotype, a multicellular behavior in *Enterobacteriaceae*. *Cell Mol. Life Sci.* 62 1234–1246. 10.1007/s00018-005-4557-x 15818467 PMC11139082

[B88] Ruzzi M. Aroca R. (2015). Plant growth-promoting rhizobacteria act as biostimulants in horticulture. *Sci. Hort.* 196 124–134. 10.1016/j.scienta.2015.08.042

[B89] Saikia J. Sarma R. K. Dhandia R. Yadav A. Bharali R. Gupta V. K. (2018). Alleviation of drought stress in pulse crops with ACC deaminase producing rhizobacteria isolated from acidic soil of northeast India. *Sci. Rep.* 8:3560. 10.1038/s41598-018-21921-w 29476114 PMC5824784

[B90] Saleh-Lakha S. Glick B. R. (2006). “Plant growth-promoting bacteria,” in *Modern Soil Microbiology*, eds van Elsas J. D. Jansson J. K. Trevors J. T. (Boca Raton, FL: CRC/Thomson Publishing), 503–520.

[B91] Sambrook J. Fritsch E. F. Maniatis T. (1989). *Molecular Cloning: A Laboratory Manual (No. Ed.* 2). Cold Spring Harbor, NY: Cold spring harbor laboratory press.

[B92] Sandhya V. Z. Grover M. Reddy G. Venkateswarlu B. (2009). Alleviation of drought stress effects in sunflower seedling by the exopolysacchrides producing *Pseudomonas putida* strain GAP-P45. *Biol. Fert. Soil.* 46 17–26. 10.1007/s00374-009-0401-z

[B93] Saravanan V. S. Madhaiyan M. Thangaraju M. (2007). Solubilization of zinc compounds by the diazotrophic, plant growth promoting bacterium *Gluconacetobacter diazotrophicus*. *Chemosphere* 66 1794–1798. 10.1016/j.chemosphere.2006.07.067 16956644

[B94] Schaad N. W. (1988). “Laboratory guide for identification of plant pathogenic bacteria,” in *American Phytopathological Society*, ed. Schaad N. W. (St. Paul, MN: APS Press).

[B95] Selin C. Habibian R. Poritsanos N. Athukorala S. N. P. Fermando D. de Kievit T. R. (2010). Phenazines are not essential for Pseudomonas chlororaphis PA23 biocontrol of Sclerotium sclerotiorum, but do play a role in biofilm formation. *FEMS Microbiol. Ecol.* 71 73–83. 10.1111/j.1574-694.2009.00792.x 19889032

[B96] Selvakumar G. Panneerselvam P. Ganeshamurthy A. N. (2012). “Bacterial mediated alleviation of abiotic stress in crops,” in *Bacteria in Agrobiology: Stress Management*, ed. Maheswari D. K. (Berlin: Springer-Verlag), 273–279.

[B97] Seneviratne S. I. Corti T. Davin E. L. Hirschi M. Jaeger E. B. Lehner I. (2010). Investigating soil moisture-climate interactions in a changing climate: a review. *Earth Sci. Rev.* 99 125–161. 10.1016/j.earscirev.2010.02.004

[B98] Sgherri C. Maffei M. Navari-Izzo F. (2000). Antioxidative enzymes in wheat subjected to increasing water deficit and rewatering. *J. Plant Physiol.* 157 273–279. 10.1016/S0176-1617(00)80048-6

[B99] Sharma S. Rai P. Rai S. Srivastava M. Kashyap P. L. Sharama A. (2017). “Genomic revolation in crop disease diagnosis: a review,” in *Plants and Microbes in an Ever Changing Environment*, ed. Singh S. S. (Hauppauge, NY: Nova Science Publishers), 257–293.

[B100] Shekhawat G. S. Chakrabarti S. K. Gadewar A. V. (1992). *Potato Bacterial Wilt in India. ICAR Technical Bulletin* 38 Shimla: Central Potato Research Institute (CPRI).

[B101] Shin S. H. Lim Y. Lee S. E. Yang N. W. Rhee J. H. (2001). CAS agar diffusion assay for the measurement of siderophores in biological fluids. *J. Microbiol. Methods* 44 89–95. 10.1016/S0167-7012(00)00229-3 11166103

[B102] Sommers L. E. Nelson D. W. (1972). Determination of total phosphorus in soils: a rapid perchloric acid digestion procedure. *Soil Sci Soc America J* 36 902–904. 10.2136/sssaj1972.03615995003600060020x

[B103] Teitzel G. M. Parsek M. R. (2003). Heavy metal resistance of biofilm and planktonic *Pseudomonas aeruginosa*. *Appl. Environ. Microbiol.* 69 2313–2320. 10.1128/AEM.69.4.2313-2320.2003 12676715 PMC154819

[B104] Thornley M. J. (1960). The differentiation of *Pseudomonas* from other gram-negative bacteria on the basis of arginine metabolism. *J. Appl. Bacteriol.* 23 37–52. 10.1111/j.1365-2672.1960.tb00178.x

[B105] Timmusk S. El-Daim I. A. A. Copolovici L. Tanilas T. Kännaste A. Behers L. (2014). Drought-tolerance of wheat improved by rhizosphere bacteria from harsh environments: enhanced biomass production and reduced emissions of stress volatiles. *PLoS One* 9:e96086. 10.1371/journal.pone.0096086 24811199 PMC4014485

[B106] Timmusk S. Grantcharova N. Wagner E. G. H. (2005). *Paenibacillus polymyxa* invades plant roots and forms biofilms. *Appl. Environ. Microbiol.* 71 7292–7300. 10.1128/AEM.71.11.7292-7300.2005 16269771 PMC1287669

[B107] Ude S. Arnold D. L. Moon C. D. Timms-Wilson T. Spiers A. J. (2006). Biofilm formation and cellulose expression among diverse environmental *Pseudomonas* isolates. *Environ. Microbiol.* 8 1997–2011. 10.1111/j.1462-2920.2006.01080.x 17014498

[B108] Ueda T. Suga Y. Yahiro N. Matsuguchi T. (1995). Remarkable N2-fixing bacterial diversity detected in rice roots by molecular evolutionary analysis of *nifH* gene sequences. *J. Bacteriol.* 177 1414–1417. 10.1128/jb.177.5.1414-1417.1995 7868622 PMC176754

[B109] Vacheron J. Desbrosses G. Bouffaud M.-L. Touraine B. Moënne-Loccoz Y. Muller D. (2013). Plant growth-promoting rhizobacteria and root system functioning. *Front. Plant Sci.* 4:356. 10.3389/fpls.2013.00356 24062756 PMC3775148

[B110] Van Oosten M. J. Di Stasio E. Cirillo V. Silletti S. Ventorino V. Pepe O. (2018). Root colonization with *Azotobacter chroococcum* 76A enhances tomato plants adaptation to salt stress under low N condition. *BMC Plant Biol.* 18:205. 10.1186/s12870-018-1411-5 30236058 PMC6149061

[B111] Vardharajula S. Ali S. Z. Reddy G. Band V. (2011). Drought-tolerant plant growth promoting *Bacillus* spp.: effect on growth, osmolytes, and antioxidant status of maize under drought stress. *J. Plant Interact.* 6 1–14. 10.1080/17429145.2010.535178

[B112] Vemanna R. S. Babitha K. C. Solanki K. Reddy A. Sarangi S. K. Udayakumar M. (2017). Aldo-keto reductase-1 (AKR1) product cellular enzymes from salt stress by detoxifying reactive cytotoxic compounds. *Plant Physiol. Biochem.* 113 177–186. 10.1016/j-plaphy-2017.02-012 28222349

[B113] Vurukonda S. S. K. Vardharajula S. Shrivastava M. SkZ A. (2016). Enhancement of drought stress tolerance in crops by plant growth promoting rhizobacteria. *Microbial. Res.* 184 13–24. 10.1016/j.micres.2015.12.003 26856449

[B114] Wang C. T. Yang W. Wang C. Gu C. Niu D. D. Liu H.-X. (2012). Induction of drought tolerance in cucumber plants by a consortium of three plant-growth promoting rhizobacterial strains. *PLoS One* 7:e52565. 10.1371/journal.pone.0052565 23285089 PMC3532358

[B115] Watson M. E. Isaac R. A. (1990). “Analytical instruments for soil and plant analysis,” in *Soil testing and plant analysis*, 3rd Edn, ed. Westermaan W. L. (Madison, WI: American Society of Agronomy), 691–740. 10.2136/sssabookser3.3ed.c26

[B116] Wezel A. Casagrande M. Celette F. Vian J. F. Ferrer A. Peigne J. (2014). Agroecological practices for sustainable agriculture: a review. *Agron. Sustain. Dev.* 34 1–20. 10.1007/s13593-013-0180-7

[B117] Williams A. Wilkinson A. Krehenbrink M. Russo D. M. Zorreguieta A. Downie J. A. (2008). Glucomannan-mediated attachment of *Rhizobium leguminosarum* to pea root hairs is required for competitive nodule infection. *J. Bacteriol.* 190 4706–4715. 10.1128/JB.01694-07 18441060 PMC2446804

[B118] Xie C. Xie Z. Xu X. Yang D. (2015). Persimmon (*Diospyros kaki* L.) leaves: a review on traditional uses, phytochemistry and pharmacological properties. *J. Ethnopharmacol.* 163 229–240. 10.1016/j.jep.2015.01.007 25637828

[B119] Yan F. Yu Y. Gozzi K. Chen Y. Guo J. H. Chai Y. (2017). Genome-wide investigation of biofilm formation in *Bacillus cereus*. *Appl. Environ. Microbiol.* 83:e561-17. 10.1128/AEM.00561-17 28432092 PMC5478996

[B120] Yang J. Kharbanda P. D. Mirza M. (2004). Evaluation of *Paenibacillus polymyxa* PKB1 for biocontrol of *Pythium* disease of cucumber in a hydroponic system. *Acta Hortic.* 635 59–66. 10.17660/ActaHortic.2004.635.7

[B121] Zhang N. Yang D. Wang D. Miao Y. Shao J. Zhou X. (2015). Whole transcriptomic analysis of the plant-beneficial rhizobacterium *Bacillus amyloliquefaciens* SQR9 during enhanced biofilm formation regulated by maize root exudates. *BMC Genom.* 16:685. 10.1186/s12864-015-1825-5 26346121 PMC4562157

[B122] Zogaj X. Nimtz M. Rohde M. Bokranz W. Römling U. (2001). The multicellular morphotypes of *Salmonella typhimurium* and *Escherichia coli* produce cellulose as the second compound of the extracellular matrix. *Mol. Microbiol.* 39 1452–1463. 10.1046/j.1365-2958.2001.02337.x 11260463

