# Supplementary figures and images for: Biofilm Producing Rhizobacteria With Multiple Plant Growth-Promoting Traits Promote Growth of Tomato Under Water-Deficit Stress
Source: Front Microbiol. 2020 Nov 26;11:542053. doi: 10.3389/fmicb.2020.542053 (PMC7727330; doi:10.3389/fmicb.2020.542053)

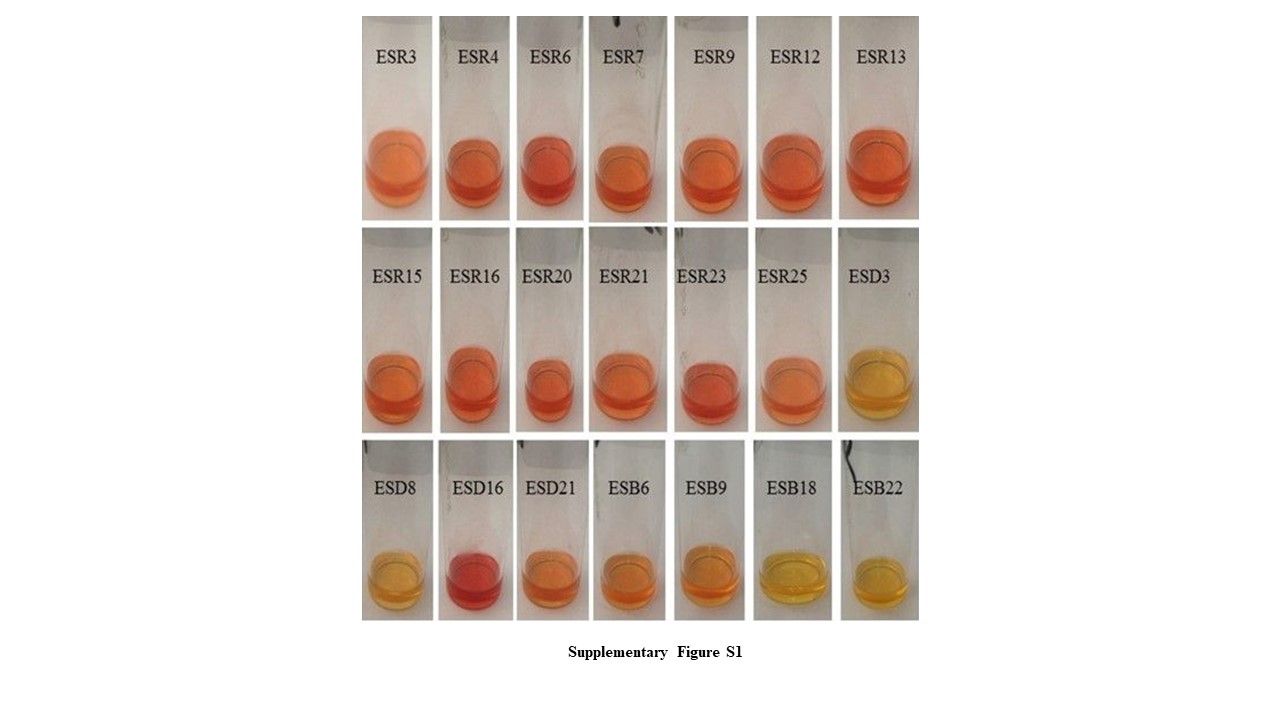

Supplement: Supplementary Figure 1 — Production of auxins by different rhizobacterial strains. Initially each rhizobacterium was grown in LB broth supplemented with 0.2% of L-tryptophan in agitating condition. After 48 h incubation, 1 mL culture was collected and centrifuged. Then 500 μL supernatant was mixed with 1 mL of Salkowski reagent. Development of pink color indicated positive for auxin related compounds including IAA production. [file Image_1.JPEG]

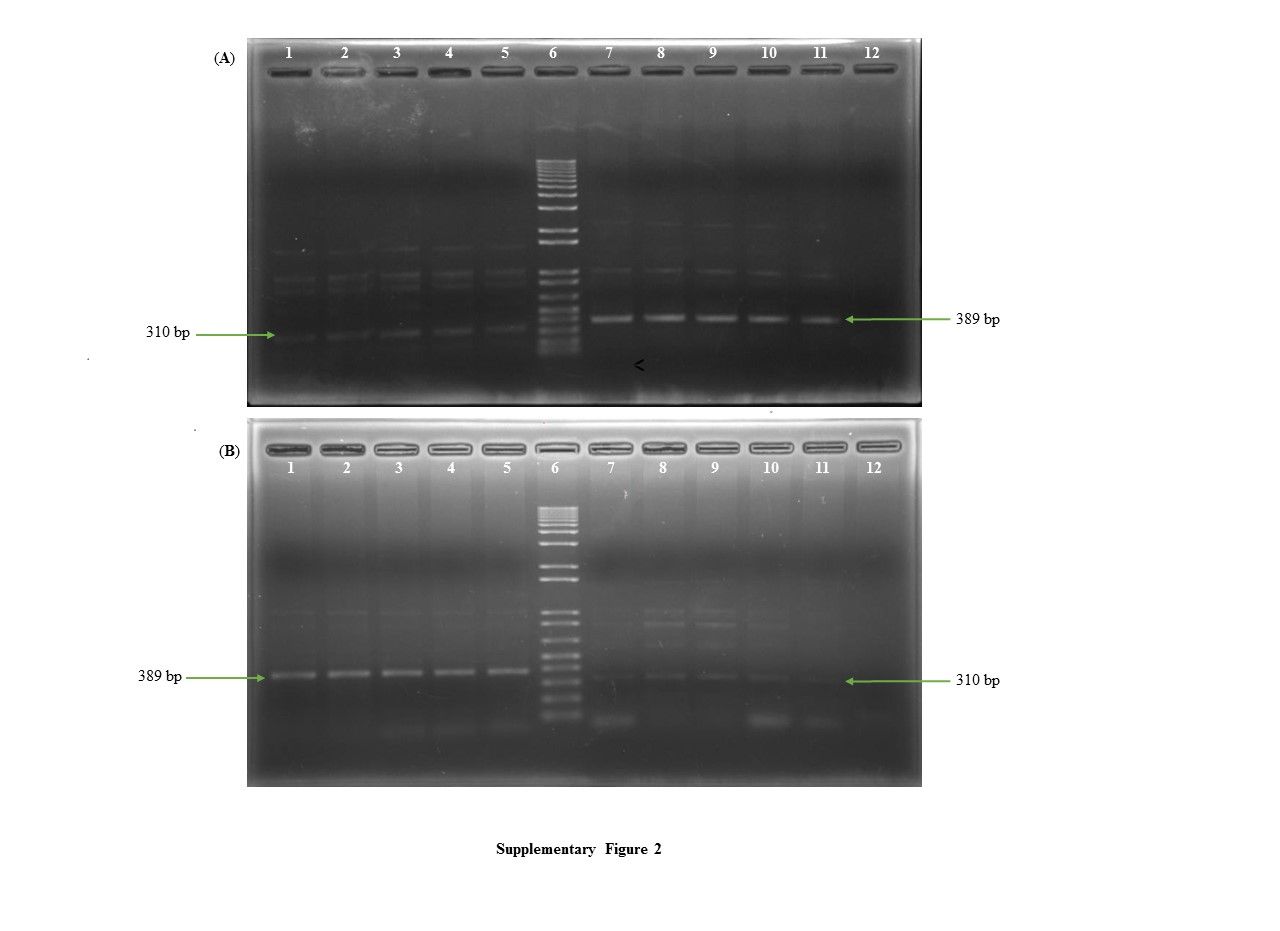

Supplement: Supplementary Figure 2 — Expression of nifH gene in different rhizobacterial strains. Primer pairs of Ueda19F and Ueda19R amplified 389 bp, while 310 pb was amplified by KAD3-F and DVV-R primer pairs. (A) Lane 1- ESR3, Lane 2- ESR4, Lane 3- ESR6, Lane 4- ESR7, Lane 5- ESR9, Lane 6- 1 kb+ ladder, Lane-7 ESD3, Lane-8 ESD8, Lane-9 ESD16 23, Lane-10 ESD21, Lane-10 ESB9, Lane-12 Negative control; Lane 1 to 5 used KAD3-F and DVV-R primers pair and lane 7-11 used Ueda19F and Ueda407R (B) Lane 1- ESR12, Lane 2- ESR13, Lane 3- ESR15, Lane 4- ESR16, Lane 5- ESR20, Lane 6- 1 kb+ ladder, Lane-7 ESR21, Lane-8 ESR23, Lane-9 ESB18, Lane-10 ESB22 72, Lane-11 ESR20, Lane-12 Negative control; Lane 1 to 5 used Ueda19F and Ueda407R primers pair and lane 7-11 used KAD3F and DVVR primers pair. [file Image_2.JPEG]

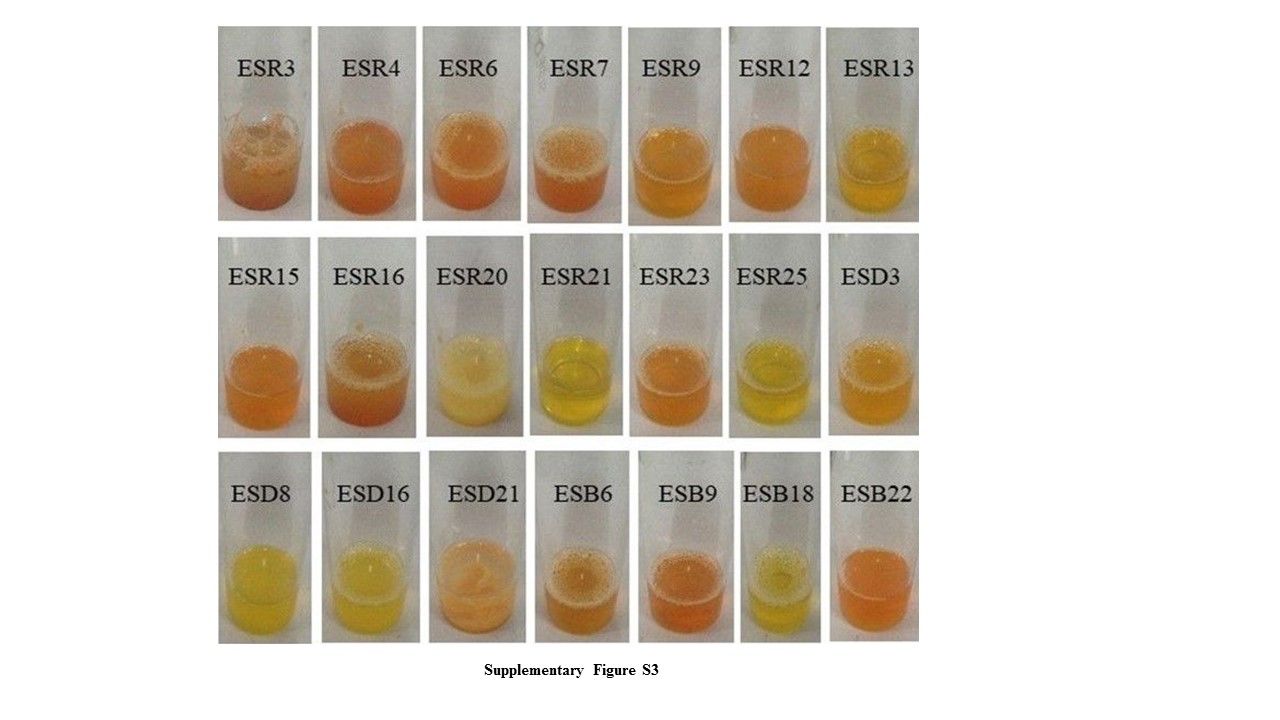

Supplement: Supplementary Figure 3 — Qualitative ammonia production by different rhizobacterial strains. The 50 μL (108 CFU mL–1) culture were inoculated in the glass test tubes containing 5 mL peptone water and incubated at 28°C for 72 h. Then 1 mL Nessler’s reagent was added. Development of yellow to brown color indicated positive results. [file Image_3.JPEG]

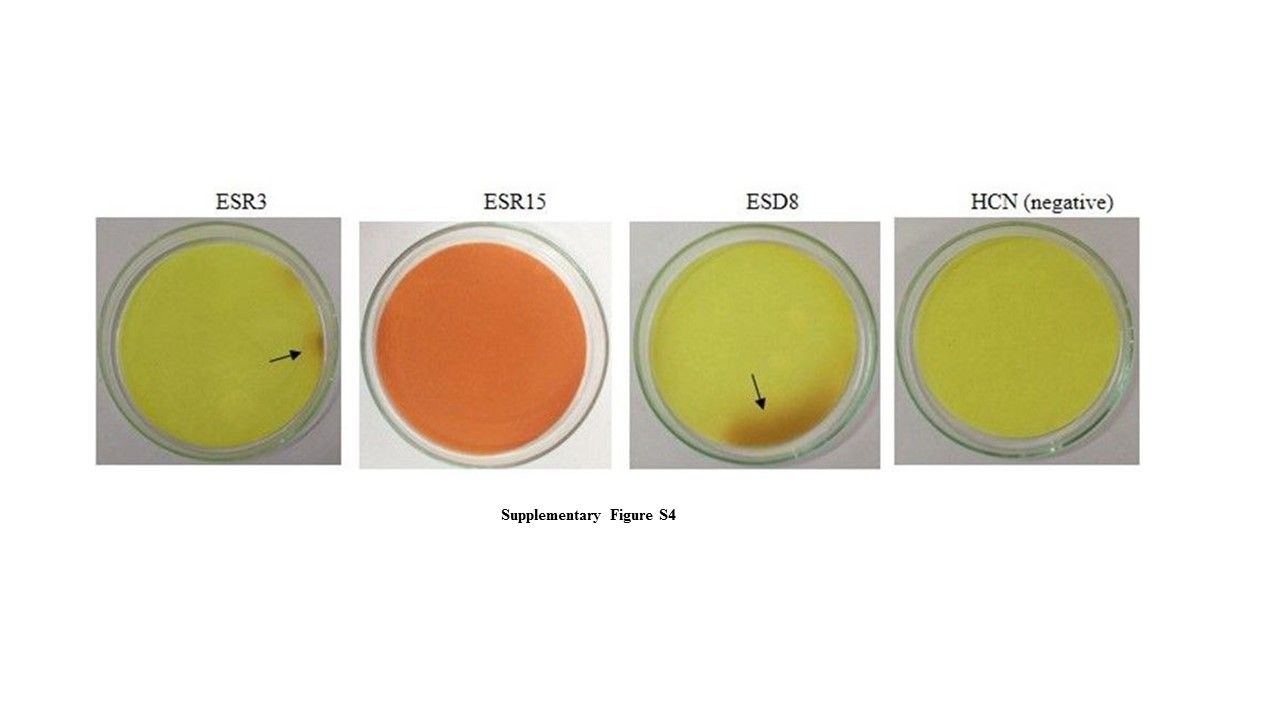

Supplement: Supplementary Figure 4 — Qualitative production of HCN. Rhizobacterial strains were streaked on LB agar plates containing 0.45% glycine. Then filter papers dipped in alkaline pirate solution and placed on the lids of petri plates, sealed with parafilm and incubated at 28°C for 24 h. Positive HCN production resulted in a color development from yellow to reddish-brown. [file Image_4.JPEG]

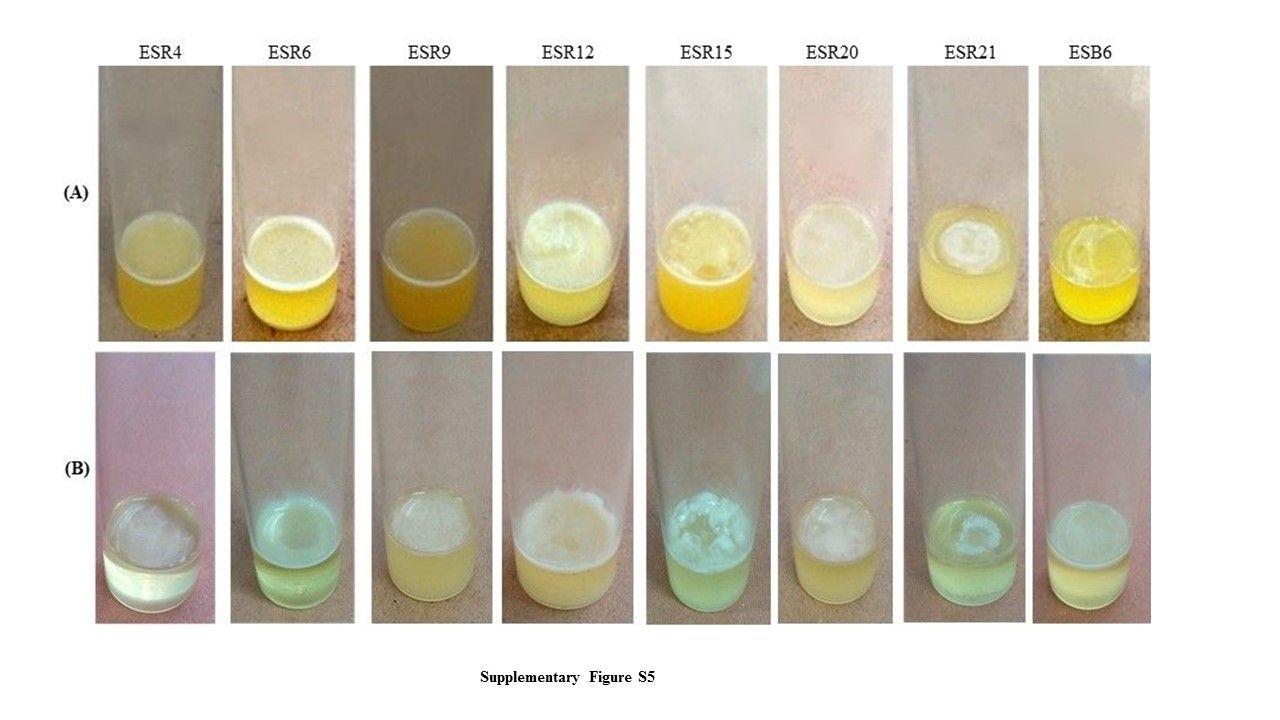

Supplement: Supplementary Figure 5 — Production of biofilms by some selected rhizobacterial strains after 72 h incubation at 28°C in stationary condition. Biofilm formation on SOBG without 25% PEG 6000 (A) and SOBG with 25% PEG 6000 (B). [file Image_5.JPEG]
